# Direct Air Capture of CO_2_ Using Amine/Alumina Sorbents at Cold Temperature

**DOI:** 10.1021/acsenvironau.3c00010

**Published:** 2023-06-29

**Authors:** Pranjali Priyadarshini, Guanhe Rim, Cornelia Rosu, MinGyu Song, Christopher W. Jones

**Affiliations:** School of Chemical & Biomolecular Engineering, Georgia Institute of Technology, 311 Ferst Drive, Atlanta, Georgia 30332-0100, United States

**Keywords:** direct air capture (DAC), adsorption, amine, alumina, carbon dioxide

## Abstract

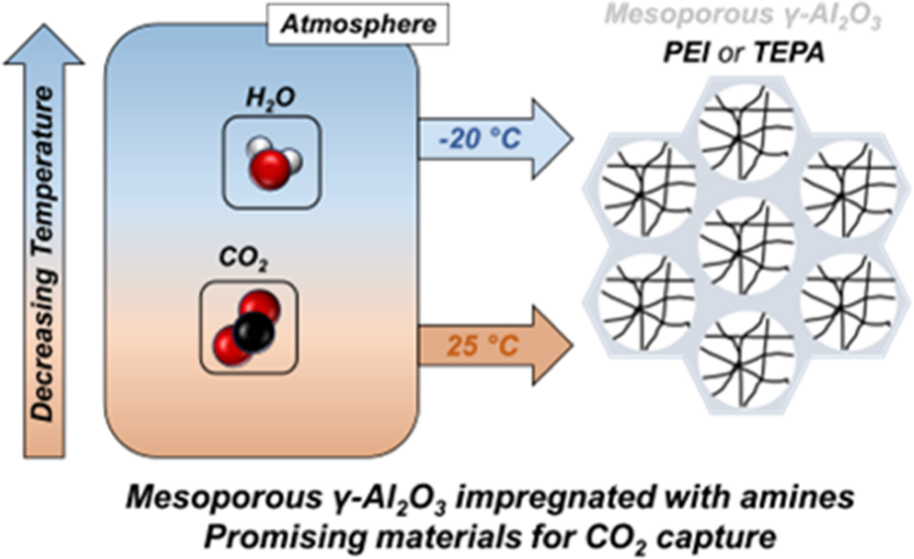

Rising CO_2_ emissions are responsible for increasing global temperatures causing climate change. Significant efforts are underway to develop amine-based sorbents to directly capture CO_2_ from air (called direct air capture (DAC)) to combat the effects of climate change. However, the sorbents’ performances have usually been evaluated at ambient temperatures (25 °C) or higher, most often under dry conditions. A significant portion of the natural environment where DAC plants can be deployed experiences temperatures below 25 °C, and ambient air always contains some humidity. In this study, we assess the CO_2_ adsorption behavior of amine (poly(ethyleneimine) (PEI) and tetraethylenepentamine (TEPA)) impregnated into porous alumina at ambient (25 °C) and cold temperatures (−20 °C) under dry and humid conditions. CO_2_ adsorption capacities at 25 °C and 400 ppm CO_2_ are highest for 40 wt% TEPA-incorporated γ-Al_2_O_3_ samples (1.8 mmol CO_2_/g sorbent), while 40 wt % PEI-impregnated γ-Al_2_O_3_ samples exhibit moderate uptakes (0.9 mmol g^–1^). CO_2_ capacities for both PEI- and TEPA-incorporated γ-Al_2_O_3_ samples decrease with decreasing amine content and temperatures. The 40 and 20 wt % TEPA sorbents show the best performance at −20 °C under dry conditions (1.6 and 1.1 mmol g^–1^, respectively). Both the TEPA samples also exhibit stable and high working capacities (0.9 and 1.2 mmol g^–1^) across 10 cycles of adsorption–desorption (adsorption at −20 °C and desorption conducted at 60 °C). Introducing moisture (70% RH at −20 and 25 °C) improves the CO_2_ capacity of the amine-impregnated sorbents at both temperatures. The 40 wt% PEI, 40 wt % TEPA, and 20 wt% TEPA samples show good CO_2_ uptakes at both temperatures. The results presented here indicate that γ-Al_2_O_3_ impregnated with PEI and TEPA are potential materials for DAC at ambient and cold conditions, with further opportunities to optimize these materials for the scalable deployment of DAC plants at different environmental conditions.

## 1 Introduction

The Industrial Revolution, beginning in the late 18th century, replaced manual labor with heavy machinery, ushering in the era of fossil energy. The increasing energy demands with an exploding population accelerated the burning of fossil fuels at ever-increasing rates. The use of fossil fuels has led to an increase in greenhouse gases, especially carbon dioxide, which trap heat, causing an increase in the average global temperatures by 1.2 °C since the industrial revolution.^1,2^ Analysis from the National Oceanic and Atmospheric Administration (NOAA) shows that the average atmospheric CO_2_ concentration reached 417 ppm in 2022, which amounts to a >2 ppm increase from the prior year.^3^ While the rise in temperature seems very small over the time period considered of 170 years (1850–2020), such a small rise in temperature has led to severe changes in weather patterns (increase in frequency and strength of storms, prolonged heatwaves, greater occurrences of forest fires, floods, etc.)^4−7^ that have negatively impacted plant and animal life.^8^ Global climate models suggest that the average global surface temperature increase needs to be limited to <2 °C by the end of the century to ameliorate the most deleterious effects of climate change.^9,10^

While significant efforts are being directed toward lowering our reliance on fossil fuels, such as the gradual shift toward renewable sources of energy, these strategies only reduce the rate of increase of CO_2_ concentration in the atmosphere. Deployment of negative emissions technologies (NETs), methods that can remove excess CO_2_ in the air, is needed to meet the goal of reducing the rate of increase of global temperatures by addressing historical emissions.^11−13^ Within this context, the capture of CO_2_ directly from air, known as direct air capture (DAC), has gained significant traction recently, and a high volume of research is being conducted across the world to design and optimize sorbents that capture CO_2_ selectively from the atmosphere with a high uptake, rapid kinetics, and sufficient stability or lifetime.^14−20^

The ideal CO_2_ sorbent should exhibit fast kinetics and high selectivity toward CO_2_ from the CO_2_-lean atmosphere (∼420 ppm CO_2_). Hence, CO_2_ should bind strongly to the adsorbent but not so effectively as to require regeneration at temperatures or conditions that will be cost-prohibitive.^21,22^ Amine-based porous adsorbents have shown potential owing to their high CO_2_ uptake capacities and lower temperature requirements for regeneration, which translates to lower energy and hence lower capital requirements.^16,19,23−26^ However, most studies have primarily focused on studying these materials at 25 °C and higher.^27−37^ Additionally, relatively few studies take into account the effect of water vapor in the atmosphere while evaluating the performance of the sorbents. Temperatures vary significantly across the globe (from −30 to 30 °C), and ∼70% of the world has an average temperature lower than 25 °C.^38,39^ Studies focusing on cooler conditions (<25 °C)^40,41^ are few and far between, which precludes informed operation in a large geographical area. Further, air at lower temperatures contains much less moisture compared to higher temperatures, which may open use of classes of materials that have not been previously seriously considered because of their poor behavior in the presence of moisture (e.g., certain MOFs,^42^ zeolites^20,43^) since these materials may not be as negatively affected by the presence of the small amounts of moisture in air at lower temperatures. Hence, studying the behavior of materials at lower temperatures will aid the widespread deployment of direct air capture plants. A recent study demonstrated that lower ambient temperatures may be preferred for DAC operations to lower the environmental impact and energy consumption.^44^ Our group recently showed that poly(ethyleneimine) (PEI)- and tetraethylenepentamine (TEPA)-incorporated MIL-101(Cr) metal–organic frameworks (MOFs) show promising uptakes and initial stabilities at cold conditions.^40^

While MIL-101(Cr)-based amine sorbents show promise for capturing CO_2_ from air, MIL-101(Cr) is not a commercially available material and its production needs to be scaled up for it to become a candidate material for DAC deployment. Alternatively, using commercially available porous materials as supports can eliminate the cost and complexity associated with synthesizing a complicated support. γ-Al_2_O_3_ is a commonly used support for heterogeneous catalytic reactions^45^ and has been investigated for CO_2_ capture as well,^46,47^ though not as extensively as other oxide supports such as silica. Previous research from our group has shown that different forms of Al_2_O_3_ show CO_2_ capacities of ∼2 mmol g^–1^ at 30 °C and are stable on exposure to steam-stripping conditions,^46−48^ which is one set of favored desorption conditions. These preliminary results and the commercial availability of alumina make it an important support material to assess under sub-ambient conditions.

Here, we investigate the effect of amine loading, adsorption temperature, and humidity on the CO_2_ adsorption performance of branched PEI- and TEPA-impregnated γ-Al_2_O_3_ sorbents at 25 and −20 °C. We will refer to 25 °C as ambient and −20 °C as sub-ambient temperature in this work, similar to our previous studies where we defined sub-ambient as below typical indoor laboratory temperature of 20 °C.^40,43^ Under dry conditions and at both −20 and 25 °C, both 20 and 40 wt % TEPA-impregnated γ-Al_2_O_3_ show high CO_2_ uptakes (>1 mmol CO_2_/g sorbent). The trends for CO_2_ uptakes at −20 °C are dissimilar to those observed at 25 °C because at such low temperatures, the arrested motion of amine chains can induce pore-blocking, convoluting the performance of the sorbents. Adsorption uptake profiles showed that higher weight loading amine samples showed an initial rapid CO_2_ uptake followed by a slow approach to equilibrium due to the high pore filling at the higher amine content. Initial adsorption rates at −20 °C were not significantly lower than those at 25 °C. Finally, the presence of water vapor improved the performance of both the PEI- and TEPA-infused γ-Al_2_O_3_ samples and the sorbents were stable under temperature swing adsorption–desorption conditions. These results indicate that amine-incorporated γ-Al_2_O_3_ adsorbents are promising candidates that can potentially be employed across a large range of operating conditions for DAC operation.

## 2 Materials and Methods

### 2.1 Material Preparation

Mesoporous γ-Al_2_O_3_ (particle size ∼6.9 μm, pore size (mode) ∼16.1 nm)^49^ was sourced commercially from Global Thermostat, LLC. Branched poly(ethyleneimine) (PEI-800, *M*_w_ ∼800, *M*_n_ ∼600 Sigma-Aldrich), tetraethylenepentamine (TEPA, Sigma-Aldrich), spermine (>97%, Sigma-Aldrich), and spermidine (>99%, Sigma-Aldrich) were incorporated into the pores of alumina by physical impregnation, as described elsewhere.^40^ Briefly, γ-Al_2_O_3_ in a round-bottom flask was first activated by evacuating the flask, heating to 80 °C, and holding at 10 mTorr and 80 °C overnight. The activated alumina was then dispersed in 25 mL of methanol by sonication to form a suspension. Separately, a requisite amount of amine (based on the desired weight loading) was dissolved in 5 mL of methanol and stirred for 15 min to dissolve completely. The amine solution was then added to the Al_2_O_3_ suspension and stirred overnight at room temperature. The methanol was removed by rotary evaporation, and the resulting solid was dried under ∼10 mTorr vacuum at 80 °C (PEI) or 60 °C (TEPA, spermine, or spermidine) overnight to yield the amine-incorporated γ-Al_2_O_3_ sorbents.

### 2.2 N_2_ Physisorption

N_2_ physisorption experiments were conducted with a surface area and porosity (SAP) system (autosorb iQ/Quantachrome). Powder samples (75–100 mg) were activated at 60 °C (TEPA) and 80 °C (PEI) under vacuum for 4 h before the measurement. The BET surface area was estimated using the N_2_ physisorption data in the *P/P*_0_ range of 0.05–0.2. Pore volumes were estimated at the partial pressure of 0.995.

### 2.3 TGA Combustion Experiments

Burnout experiments were conducted to determine the content of amines in the pores of the γ-Al_2_O_3_ support on a Q500 TA Instruments TGA. Powder samples (10–20 mg) were loaded onto the platinum sample pans and heated from room temperature to 700 °C under N_2_ gas (Airgas, 99.998% purity) at 10 °C min^–1^, and the weight of the sample was recorded. Pristine γ-Al_2_O_3_ samples were also measured as a control, and the data beyond 150 °C were used to determine the organic content of the samples.

### 2.4 CO_2_ Adsorption Isotherms

The equilibrium CO_2_ adsorption isotherms of amine-incorporated γ-Al_2_O_3_ samples were measured under dry ambient temperature (25 °C) and sub-ambient (−20 °C) conditions in the SAP system (autosorb iQ/Quantachrome). Powder samples (∼80 to 100 mg) were activated at 60 °C (TEPA, spermine or spermidine sorbents) or 80 °C (PEI-impregnated materials) under vacuum for 4 h before the CO_2_ adsorption experiment. The gases used during the experiments were He (Airgas, UHP) and CO_2_ (Airgas, Bone dry). An equilibration interval of 5 min was used for all of the CO_2_ pressures. During equilibration, the cell pressure was checked every 1 min and compared until the pressure in the cell was within the *P* tolerance (manufacturer tolerance setting 0). If the cell pressure dropped below the lower limit of the *P*/*P*_0_ tolerance, the data point was then stored. The total measurement times varied between 10 and 30 h depending on the amine content (10, 20, 40 wt %) and the adsorption temperature.

### 2.5 CO_2_ Adsorption Kinetics and Temperature-Programmed Desorption (TPD)

The CO_2_ uptakes of amine-impregnated γ-Al_2_O_3_ sorbents at 400 ppm CO_2_ (balance He) were also measured gravimetrically by a thermogravimetric analysis/differential scanning calorimetry (TGA/DSC) system (STA 449 F3 Jupiter/NETZSCH) under dry conditions at 25 and −20 °C. Amine-impregnated samples (∼20 mg) were activated at 60 °C (TEPA-impregnated samples) or 80 °C (PEI-impregnated materials) by heating samples at 10 °C min^–1^ under flowing He (90 mL min^–1^) for 4 h, followed by thermal equilibration at adsorption temperature conditions (25 or −20 °C) for 1 h under He. The sample was then exposed to 400 ppm of CO_2_ (Airgas, 90 mL min^–1^) for 12 h, and changes in mass was recorded. After 12 h of the adsorption step, the samples were flushed with He (90 mL min^–1^) for 1 h. The samples were slowly heated at 0.5 °C min^–1^ to 110 °C and then held at 110 °C for 2 h to completely desorb the CO_2_. The CO_2_ desorption profile was recorded by an infrared gas analyzer (LI-COR, LI-840) connected to the outlet of the TGA/DSC.

### 2.6 Temperature Swing Adsorption–Desorption Cyclic Tests

Temperature swing adsorption–desorption cycles were performed for up to 10 cycles on the TGA/DSC system described above. The desorption temperature was selected based on the desorption profile from the TPD experiments. Sorbents were regenerated by heating the samples at 10 °C min^–1^ under flowing He (90 mL min^–1^) to the desorption temperature and holding at that temperature for 2 h after adsorption (held at adsorption temperature for 2 h instead of 12 h, unlike the TPD experiments).

### 2.7 Humid Experiments

Dry and humid breakthrough experiments were performed in a custom-built fixed bed reactor equipped with a cold box (Espec, SH-642). The requisite length of the inlet and the fixed bed reactor (stainless steel, 0.63 cm O.D., 10 cm length) were enclosed inside the cold box. Powder samples (55–65 mg) were loaded into the reactor, sandwiched between glass wool on either side. Samples were activated under a dry N_2_ flow (50 mL min^–1^) for 3 h in the cold box. The inlet gas stream was then switched to dry or humid (70% RH at −20 or 25 °C) 400 ppm of CO_2_ (50 mL min^–1^) gas balanced in N_2_. In the case of the humid experiments, the humidity of the inlet gas stream was controlled by bubblers whose temperature was controlled by a water bath. The temperature of the water bath with the bubblers was set to 20 °C, and the flow rate of the inlet gas (N_2_ or CO_2_) was controlled by mass flow controllers (Alicat, MC series). The outlet from the bubbler containing the humid stream was mixed with a dry stream of the same gas. The flow rates of the humid and the dry stream were controlled to yield a humid stream at the target humidity levels that was then introduced into the fixed bed. Before the adsorption step, the sorbents were prehumidified by introducing wet N_2_ gas (70% RH at −20 or 25 °C) before exposing the sample to humid CO_2_ gas. The prehumidification process was conducted at the adsorption temperature until the water concentration of the outlet gas stream reached that of the inlet gas stream (70% RH). During the breakthrough experiments, the CO_2_ and H_2_O concentrations of the outlet gas stream were continuously measured every second by an infrared gas analyzer (LI-840/LI-COR). After the samples were saturated, humid CO_2_ flow was turned on until the outlet CO_2_ concentration became 400 ppm (i.e., same as the inlet concentration).

## 3 Results and Discussion

### 3.1 CO_2_ Adsorption under Dry Conditions

**Figure 1:**
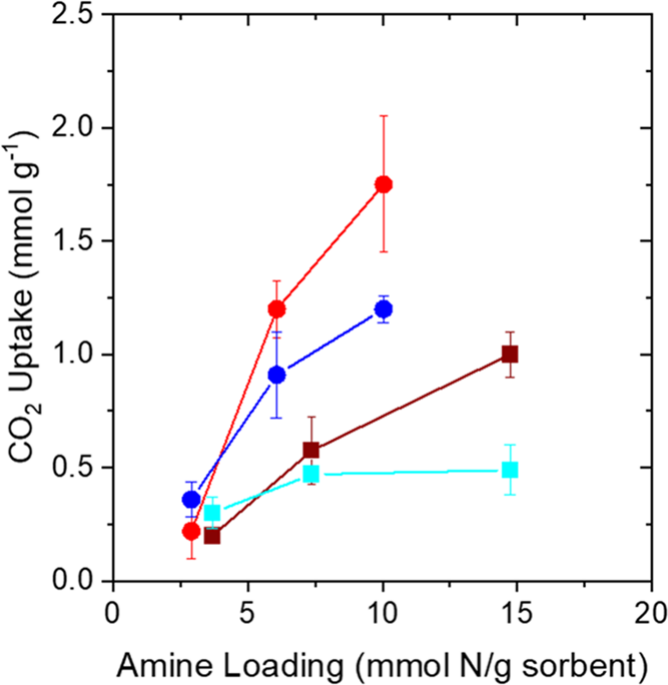
CO_2_ capacities as a function of amine loading (PEI (■) and TEPA (●)) at 25 °C (red) and −20 °C (blue) at 400 ppm CO_2_ (balance He).

The physical properties of the sorbents relevant to this study are summarized in Table S1. While linear PEI is a straightforward counterpart to TEPA, a mostly linear oligomer, branched PEI (bPEI) is used in this study because of the ubiquitous nature of bPEI in an overwhelming majority of DAC studies, making bPEI the benchmark material against which similar materials can be compared. Linear PEI has shown similar performance to branched PEI in different studies; however, challenges with linear PEI suggest it to be a less effective active phase.^50^Figure 1 shows the CO_2_ capacities of the γ-Al_2_O_3_ impregnated with different amounts of PEI and TEPA (10, 20, and 40 wt %) at 25 and −20 °C at 400 ppm CO_2_. At 25 °C, CO_2_ capacities increase for both TEPA and PEI sorbents with an increase in the amine content, as seen in Figure 1 and Table 1. For PEI-loaded samples, capacities increased from 0.2 mmol g^–1^ for 10 wt % PEI to 0.58 mmol g^–1^ for 20 wt % and 1 mmol g^–1^ for 40 wt % PEI. For the case of TEPA-impregnated γ-Al_2_O_3_, the CO_2_ uptake increased from 0.22 mmol g^–1^ for the 10 wt % sample to 1.2 mmol g^–1^ for 20 wt % to 1.8 mmol g^–1^ for the 40 wt % TEPA sorbent at 25 °C. An increase in amine loading contributes more amine sites for CO_2_ molecules to adsorb; hence, the CO_2_ uptake increases. Increased loadings of amines for both TEPA and PEI beyond 40 wt % (pore filling of 65 and 72%, respectively) were difficult to achieve because the samples could not be dried completely at the higher loadings and significant amounts of amines clung to the outer surface of the γ-Al_2_O_3_ support even after prolonged exposure to vacuum and high temperature. The CO_2_ uptakes for TEPA-incorporated materials are higher than the PEI ones by a factor of ∼2 for the 40 and 20 wt % samples even for the PEI samples that contain more moles of nitrogen, as seen in Figure 1. However, PEI possesses primary, secondary, and tertiary amines (Scheme S1 for structure) in the theoretical ratio of 1:2:1 (actual ratios may vary slightly depending on the manufacturer and batch),^51^ and tertiary amines are not suitable for adsorbing CO_2_ under dry conditions.^52^ TEPA, in contrast, contains only primary and secondary amines. So, not all amine sites in PEI are “active” or available for CO_2_ adsorption under these conditions.^53^ Moreover, the shorter chains of TEPA may facilitate the diffusion of CO_2_ better due to higher chain mobility compared to PEI, whose longer chains may inhibit effective CO_2_ diffusion due to chain entanglement,^54,55^ making TEPA-impregnated Al_2_O_3_ a better candidate for CO_2_ capture at 25 °C under dry conditions. The amine efficiencies (Table 1) for the PEI samples are much lower than the ones for TEPA samples for all loadings. The highest amine efficiency (∼0.2) was observed for the 20 wt % TEPA sorbents. Unlike CO_2_ capacities, the amine efficiencies do not increase linearly with an increase in amine loadings for both PEI and TEPA (Table 1). The amine efficiencies plateau or decrease when the amine loadings are increased from 20 to 40 wt %. The nonlinear change in the amine efficiencies indicates that there is ineffective utilization of amine sites as the pore filling increases (Table S1), likely arising due to enhanced barriers to CO_2_ diffusion with the increase in amine loadings. Another contributing factor for the lower amine efficiency at higher loadings could be the reduced availability of paired amine sites required to form carbamates since the concentration of isolated amine sites increases with amine loading.^56,57^

**Table 1:** Summary of Volumetric CO_2_ Uptakes and Amine Efficiencies at 25 and −20 °C

	CO_2_ capacity (mmol CO_2_) (g sorbent)^−1^	amine efficiency (mol CO_2_) (mol N)^−1^	CO_2_ capacity (mmol CO_2_) (g sorbent)^−1^	amine efficiency (mol CO_2_) (mol N)^−1^
sample	25 °C		–20 °C	
40 wt % PEI-Al_2_O_3_	1 ± 0.1	0.07	0.49 ± 0.11	0.03
20 wt % PEI-Al_2_O_3_	0.58 ± 0.08	0.08	0.47 ± 0.03	0.06
10 wt % PEI-Al_2_O_3_	0.2 ± 0.03	0.05	0.3 ± 0.07	0.08
40 wt % TEPA-Al_2_O_3_	1.8 ± 0.3	0.17	1.2 ± 0.06	0.12
20 wt % TEPA-Al_2_O_3_	1.2 ± 0.13	0.20	0.9 ± 0.2	0.15
10 wt % TEPA-Al_2_O_3_	0.22 ± 0.07	0.075	0.36 ± 0.08	0.12

For this paper, the sub-ambient temperature chosen was −20 °C since the temperature is at the lower extreme of global temperatures and adsorption properties can be expected to change significantly at such low temperatures. Figure 1 shows the CO_2_ capacities decrease for the higher two amine loadings (20 and 40 wt %) when the adsorption temperature is lowered from 25 to −20 °C, while the capacities marginally increase at the 10 wt % amine loading. At −20 °C, the volumetric CO_2_ capacity for 10 wt % PEI-impregnated Al_2_O_3_ is 0.3 mmol g^–1^ (∼10% rise from 25 °C) which increases to 0.47 mmol g^–1^ for the 20 wt % PEI material (∼20% decrease from 25 °C). However, as the loading is further increased to 40%, the CO_2_ uptakes remain almost the same (0.49 mmol g^–1^), which is a departure from the trends observed at 25 °C. Similarly, for TEPA, as the loading increases from 10 to 20 wt %, the CO_2_ capacities increase from 0.36 to 0.9 mmol g^–1^. The CO_2_ uptake increases by 60% for 10 wt % TEPA when the adsorption temperature is reduced from 25 to −20 °C but reduces by ∼12% for the 20 wt % TEPA sorbent. Unlike PEI, the CO_2_ capacities also increase from 0.9 to 1.2 mmol g^–1^ when the TEPA loading is increased from 20 to 40 wt % at −20 °C. However, the CO_2_ capacities increased by 50% from 20 to 40 wt % TEPA at 25 °C, compared to only 33% at −20 °C. The differences in the extent of increase at different temperatures show that the amine loading is not the singular factor affecting the CO_2_ capacities. At the significantly colder temperature of −20 °C, the polyamine chains can have their motions arrested (sometimes stated as freezing), leading to lower CO_2_ accessibility to the bulk of the oligoamine or polyamine.^58^ The lower chain mobility inhibits the diffusion of CO_2_ through the pores of the γ-Al_2_O_3_ leading to lowered CO_2_ capacities at low temperatures and high amine loadings. When the amine loadings are decreased to 10 wt %, the CO_2_ capacities at −20 °C become larger than those at 25 °C for both PEI and TEPA samples. The reversal of trends indicates that at the lower loadings, the diffusion limitations are lower which allows access to more amine sites for CO_2_ adsorption. At lower amine loadings, there also may be a greater degree of physisorption at nonamine sites. The 20 wt % TEPA sample outperformed all of the samples with the highest amine efficiency (0.15) at −20 °C. The high amine efficiency of 20 wt % TEPA at both 25 and −20 °C under dry conditions indicates that it is a promising material for CO_2_ capture in multiple conditions.

### 3.2 CO_2_ Adsorption and Desorption Behavior under Dry Conditions Measured by TGA/DSC

**Figure 2:**
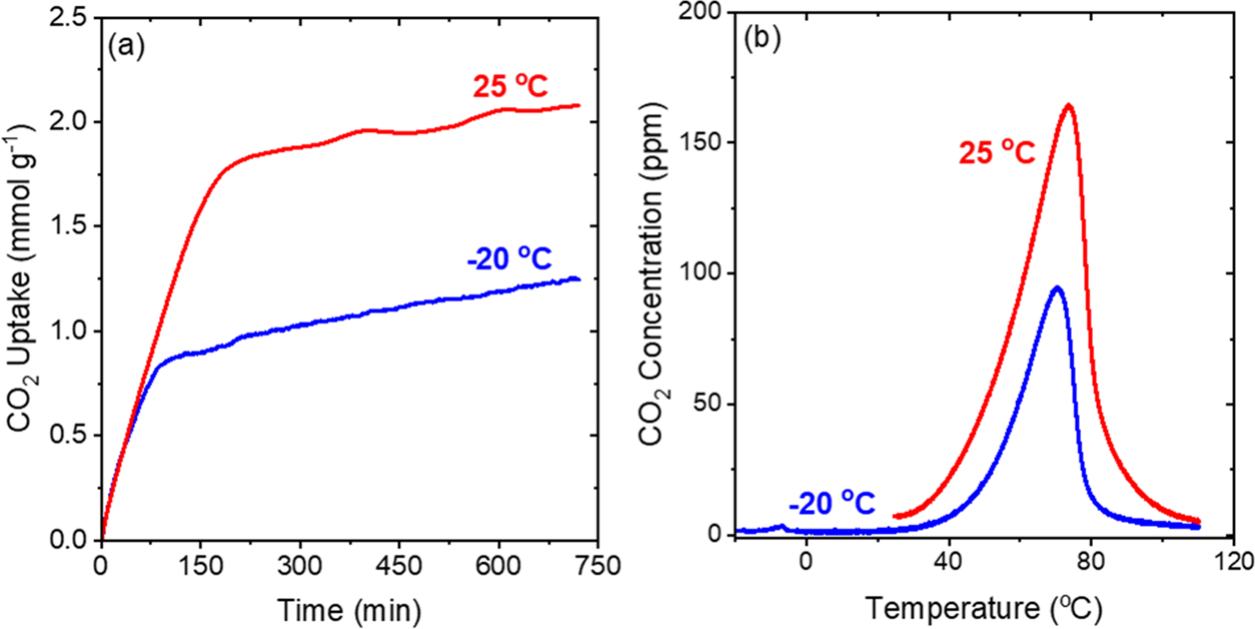
(a) CO_2_ adsorption profile of 40 wt % TEPA-impregnated γ-Al_2_O_3_ at 25 and −20 °C at 400 ppm CO_2_ (balance He). (b) CO_2_-TPD profiles after adsorption at 25 and −20 °C (measured by TGA/DSC).

**Figure 3:**
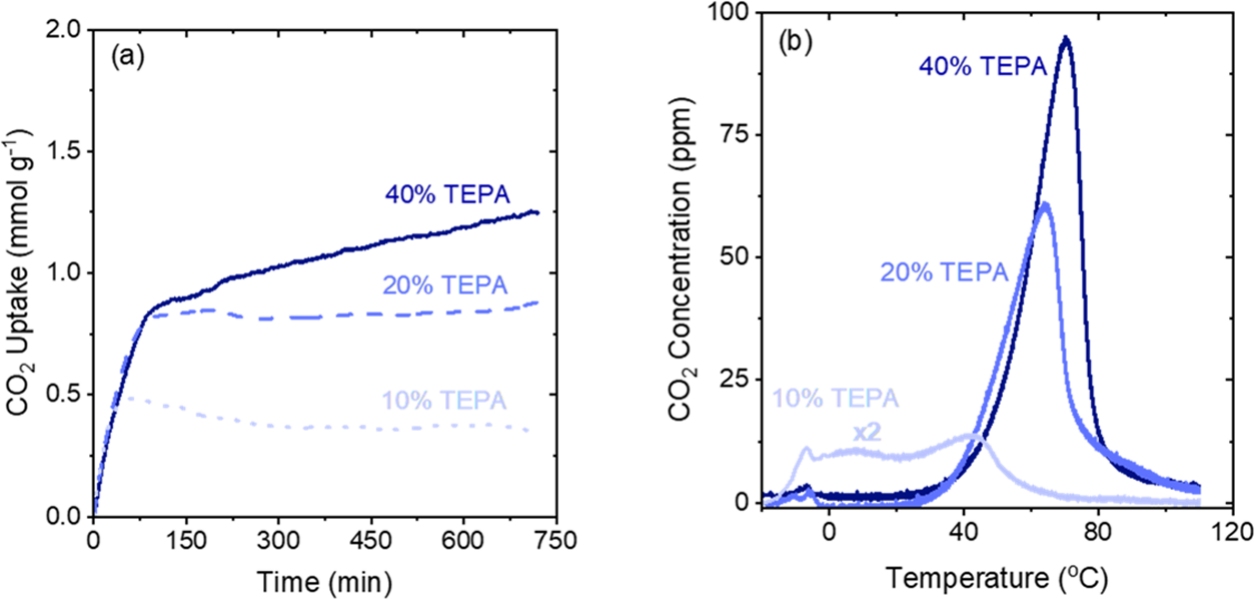
(a) CO_2_ uptake profile of 40, 20, and 10 wt % TEPA-impregnated γ-Al_2_O_3_ at −20 °C and 400 ppm CO_2_ (balance He). (b) CO_2_-TPD profiles after CO_2_ adsorption at −20 °C (measured by TGA/DSC).

**Figure 4:**
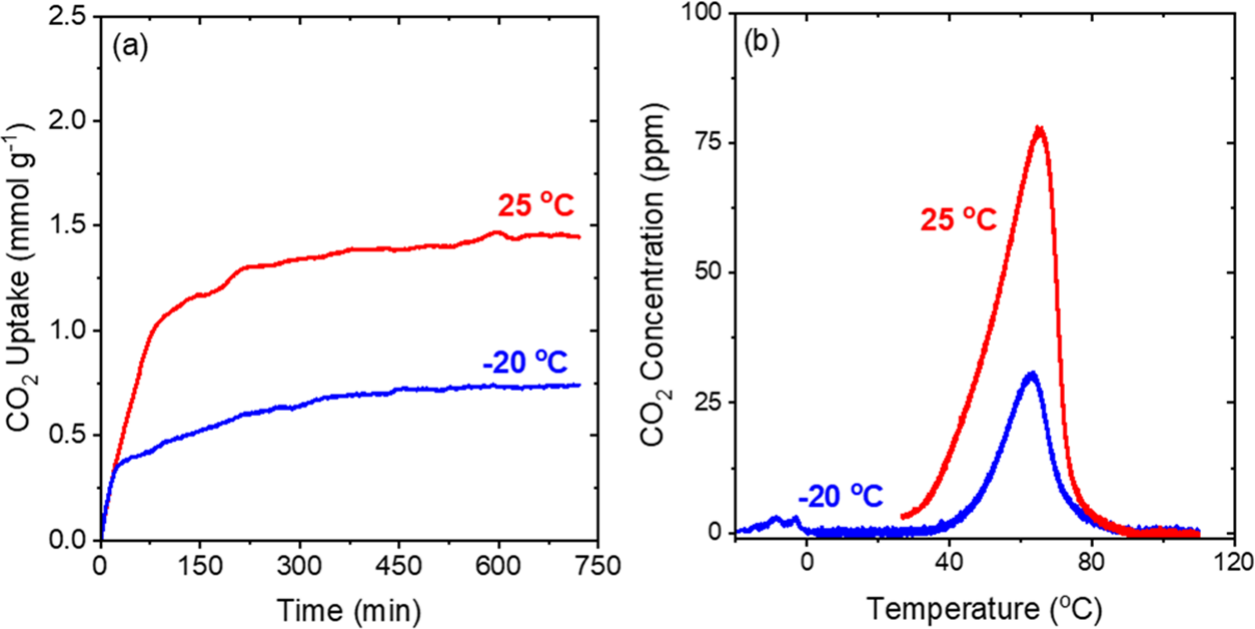
(a) CO_2_ uptake profile of 40 wt % PEI-impregnated γ-Al_2_O_3_ at 25 and −20 °C at 400 ppm CO_2_ (balance He). (b) CO_2_-TPD profiles after adsorption at 25 and −20 °C (measured by TGA/DSC).

Apart from adsorption performance, it is also important to understand the mechanisms through which CO_2_, amines, and the pore walls of γ-Al_2_O_3_ interact and what factors influence these interactions in different sorbents. Prior work using TPD studies has helped to characterize the strength of CO_2_–sorbent interactions based on CO_2_ desorption temperatures.^40,59,60^Figure 2a shows the CO_2_ adsorption profiles for 40 wt % TEPA-impregnated γ-Al_2_O_3_ samples at 25 and −20 °C. The CO_2_ adsorption profiles, especially at −20 °C, can be wavy (Figures 2–4, S2, and S3) due to the injection of pulses of liquid nitrogen intermittently to maintain the temperature in the TGA/DSC system. The CO_2_ uptakes rapidly increase in the beginning for both the temperatures and slow down after 75–150 min of exposure to 400 ppm CO_2_. However, even after 12 h, the sample does not reach a steady-state value at both temperatures, indicating that there may be some pore-blocking due to the increased pore filling at the high TEPA loading that slows the diffusion of CO_2_ through the pores.^61^ The initial rates of adsorption are similar at both 25 °C and −20 °C (Table S2). The CO_2_ capacities obtained after 720 min of adsorption were 2.1 mmol g^–1^ at 25 °C and 1.25 mmol g^–1^ at −20 °C. The decrease in the CO_2_ capacities with the decrease in temperature matches the trend seen in Figure 1 (volumetric CO_2_ capacities). However, the values obtained by the gravimetric method are ∼15 to 30% higher than the volumetric uptakes, which may be due to the differences in techniques and due to the slow sorption kinetics, that prevent the achievement of a true equilibrium in almost all cases ever reported for this class of materials. Figure S2a shows that trends for 20 wt % TEPA samples are again similar to the trends observed in Figure 1 where the CO_2_ capacities decrease with temperature, however to a lesser extent than the 40 wt % sample.

Temperature-programmed desorption (TPD) measurements were performed to determine the strength of interactions between the active amine sites and the adsorbed CO_2_. The changes in the desorption profile with changes in adsorption temperature and amine content can indicate the strength of interactions between CO_2_ and amine sites. Figure 2b shows the CO_2_ desorption profile after adsorption at 25 and −20 °C for the 40 wt % TEPA- incorporated sample. Once heating of the sample commences, CO_2_ starts desorbing at ∼25 °C for both the samples and attains a maximum at ∼70 °C. The shape of the profile and the starting temperature of desorption are nearly identical for both the adsorption temperature conditions. For adsorption at −20 °C, a small peak is observed below 0 °C just as the sample starts heating after adsorption. The small peak at lower temperatures is attributed to the small quantity of CO_2_ that is weakly bound (physisorbed) to the walls of the alumina and the amine sites.^62^ The peak arising at 70 °C is assigned to CO_2_ that is strongly bound (chemisorbed) to the amines.^62^ It is evident from Figure 2b that the adsorption temperature does not significantly impact the desorption profiles, which contrasts with the results obtained for TEPA-incorporated MIL-101(Cr) samples in our earlier study where the desorption peak shifted to lower temperatures with the decrease in adsorption temperature, indicating that the temperature of adsorption is critical in determining the interaction of CO_2_ to the amines when MIL-101(Cr) was used as a support.^40^ The differences in the results using different supports (γ-Al_2_O_3_ in this study and MIL-101(Cr)) as well as the difference in CO_2_ uptake trends with different temperatures and loadings indicate that the choice of the support is crucial in determining the performance of the sorbent at a particular condition.^49^

The effect of amine loading for TEPA samples on uptake and desorption profiles is shown in Figure 3a,b. Figure 3a and Table S2 show that decreasing the TEPA content in the pores increases the initial rate of adsorption. The time taken for the samples to reach a pseudo-equilibrium capacity decreases as the amine content is reduced from 40 wt % (which never reaches a pseudo-steady state even after 720 min) to 20 wt %, which reached a pseudo-steady state in ∼75 min compared to the 10 wt % TEPA sorbent that reached pseudo-equilibrium in ∼50 min. As seen in Table S1, decreasing the TEPA loading from 40 to 10 wt % decreases the pore filling from 65 to 23% leading to an increase in free pore volume, implying that there is less pore-blocking thereby facilitating the diffusion of CO_2_.

Figure 3b shows the effect of amine content on the CO_2_ desorption profiles after adsorption at −20 °C. The 40 and 20 wt % TEPA samples both showed a small peak below 0 °C that arises due to the desorption of CO_2_ that is physisorbed on the sample. Both the higher weight loading samples showed CO_2_ desorption starting at ∼25 °C with a peak at ∼70 and 60 °C for the 40 and 20 wt % samples, respectively. On the other hand, the desorption profile for the 10 wt % sample looks distinct from the others. CO_2_ begins desorbing at −20 °C and a broad desorption peak (consisting of multiple peaks) follows as the sample was heated. The desorption peak shifts to lower temperatures as the TEPA content is decreased. While the difference in the desorption profiles between 40 and 20 wt % TEPA samples was not significant except for a small shift (∼10 °C) to lower desorption temperatures, the desorption profile of 10 wt % TEPA sample was not only shifted to lower desorption temperatures, but the profile was also significantly broader.

The lower desorption temperature indicates that the CO_2_ is weakly bound to the amine (and possibly other) sites on the 10 wt % TEPA sample and a significant fraction of the amines interact strongly with the walls of the γ-Al_2_O_3_ support. The stronger the interaction of the amine with the walls of the support, the weaker will be its interaction with CO_2_ and hence the lower the desorption temperature. Results from Figure 3b suggest that when the amine content is low, the amine interacts strongly with the walls of the porous support. Previous results for PEI-impregnated SBA-15 materials have shown that at lower loadings, PEI forms a conformal coating on the pore walls, and starts forming aggregates at higher loadings.^63−65^ Similarly, TEPA may be coating the walls of the γ-Al_2_O_3_ sample at low loading leading to strong interactions between the amine and the γ-Al_2_O_3_ walls, thus leading to weaker interactions between the amines and CO_2_. As the loading increases, TEPA may form multilayers or aggregates, separating some amines from the pore walls and lowering the average degree of interaction of amines with the pore walls. Additionally, the desorption peak for the 10 wt % TEPA sample is very broad indicating that there is heterogeneity in the interaction of CO_2_ with the amines or other, alumina-based adsorption sites. There is a distribution of CO_2_ physisorbed (as indicated by peaks present at lower desorption temperatures), and weakly chemisorbed as shown by peaks present over 30 °C. In contrast, the 20 and 40 wt % samples do not show such broad peaks, indicating that CO_2_ is primarily chemisorbed onto the amines. The stronger interactions of the amines with the CO_2_ indicate that the amines do not strongly interact with the walls of the γ-Al_2_O_3_ at high loadings.

Similar comparisons were made for the PEI-impregnated samples as well. Figures 4a and S3a show the behavior of the CO_2_ uptake profiles with time at different temperatures and amine loadings. Similar to the TEPA samples, there is no significant difference in the adsorption kinetics of 40 and 20 wt % PEI at 25 and −20 °C (Table S2). Unlike TEPA, however, the 40 wt % PEI samples do reach a pseudo-equilibrium with a capacity of ∼1.4 mmol g^–1^ at 25 °C and ∼0.75 mmol g^–1^ at −20 °C. Again, the trends are identical to those observed in Figure 1, but the absolute values of CO_2_ uptakes are 30–40% higher for the gravimetric measurements compared to the volumetric results. The 20 wt % PEI sample (Figure S3) shows a similar trend to the 40 wt % samples. The difference in CO_2_ uptakes at 25 and −20 °C is not significant, which is identical to what was observed in Figure 1.

The CO_2_ desorption profiles for 40 wt % PEI (Figure 4b) and 20 wt % PEI (Figure S3b) also follow similar trends as the TEPA samples in Figures 2b and S2b. For both 40 and 20 wt % PEI samples, after CO_2_ adsorption at 25 °C, the desorption of CO_2_ begins at ∼25 °C and shows a peak at 60 and 50 °C, respectively. The desorption peaks are present at ∼10 °C lower temperatures compared to the TEPA samples with similar amine content, suggesting that CO_2_ binds more strongly with TEPA compared to PEI. As mentioned earlier, CO_2_ can adsorb onto the amine sites via the formation of carbamic acid (favored on secondary amines) or carbamate species (favored on primary amines).^66,67^ Since PEI and TEPA contain different fractions of primary and secondary amine sites, one may expect dissimilar distributions of carbamate and carbamic acid species for both TEPA and PEI and the strength of CO_2_ bound to each amine reflects the inherent differences in their structure. The CO_2_ desorption profiles after adsorption at −20 °C also show a small peak below 0 °C that corresponds to physisorbed CO_2_. CO_2_ starts significantly desorbing at ∼40 °C, with a peak at 60 °C. The temperature of adsorption does not change the desorption profile and peak position for both the 40 and 20 wt % PEI samples.

Figure S3a,b shows the similar effect of amine content on the CO_2_ uptake and desorption profiles at −20 °C of PEI-impregnated samples as TEPA. These results (discussed in greater detail in Section S4) indicate that reminiscent of TEPA samples, PEI would also mostly coat the walls of γ-Al_2_O_3_ at lower amine loadings, thus interacting more with the walls and less with CO_2_. On the other hand, after initially coating the walls of the support, PEI likely forms multilayers of aggregates at higher amine loadings,^63^ which contain a greater fraction of free amines, thereby reducing the average degree of interaction with the walls of the support.

The similarities in the trends and desorption profiles for both PEI and TEPA samples with changing adsorption temperatures and amine loading indicate that inherently, both PEI and TEPA behave similarly when confined in the pores of γ-Al_2_O_3_, even though their molecular structures are different. The difference in structures leads to different CO_2_ uptake capacities; however, the trends across different materials and conditions remain identical. CO_2_ uptake profiles at sub-ambient conditions show that, contrary to expectations, the rate of CO_2_ uptake is not significantly impacted and these amine sorbents can be potential candidates for DAC operations at low temperatures.

### 3.3 Temperature Swing Adsorption–Desorption Cycles under Dry Conditions

The ability of a sorbent to withstand multiple cycles over many months is important to reduce the costs associated with DAC operation. Temperature swing adsorption–desorption tests are often performed to evaluate the recyclability of a sorbent. The desorption temperature has a critical impact on the cost of DAC operations, with lower energy input yielding reduced costs. Amine-based sorbents usually require desorption temperatures in the range of ∼80 to 100 °C in the absence of vacuum, which accounts for the significant energy demand of processes based on these (and other) chemisorbents, resulting in increased costs for DAC operation.^68^ Sub-ambient DAC will require a larger temperature swing range due to the low adsorption temperatures if the same regeneration conditions are deployed, which would further elevate the cost of operating DAC plants under colder conditions. Hence, it is crucial to operate the system at the optimum sorbent regeneration temperature to achieve a suitable working capacity for the sorbent alongside low energy expenditure.

**Figure 5:**
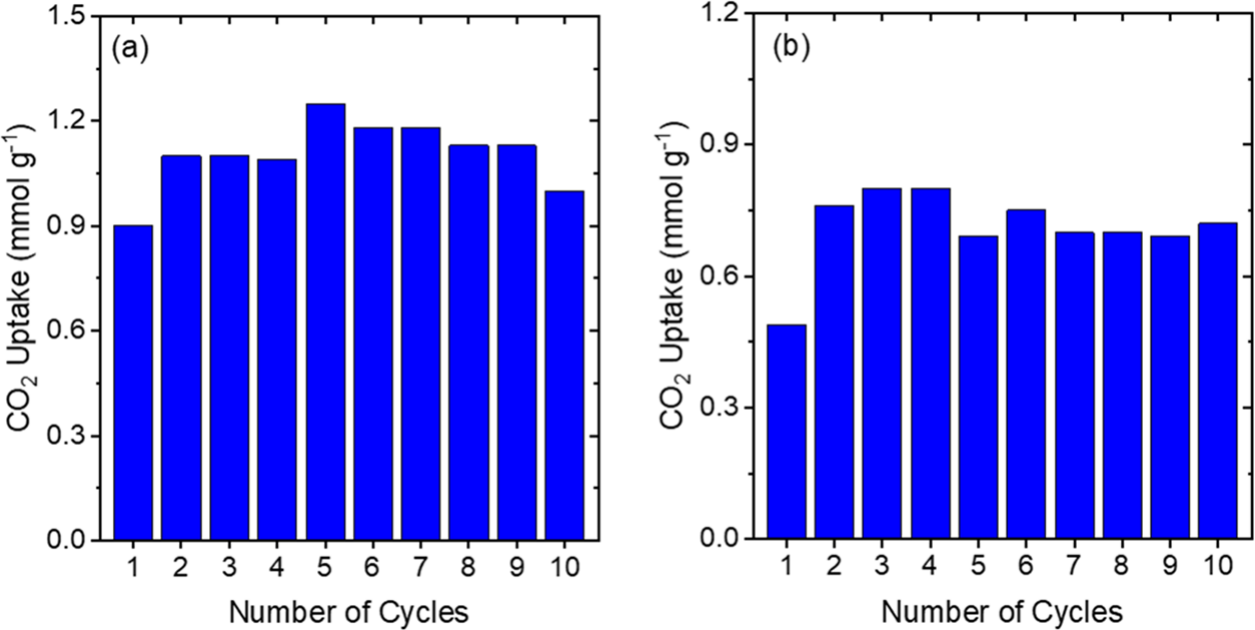
Adsorption–desorption cycles for (a) 20 wt % TEPA-impregnated γ-Al_2_O_3_ powder sorbents and (b) 20 wt % PEI-impregnated γ-Al_2_O_3_ powder sorbents over −20 °C and 400 ppm of CO_2_ (balance He).

The temperature-programmed desorption profiles were used to determine the sorbent regeneration temperatures for subsequent cyclic tests. Temperatures close to the maxima were chosen as the desorption temperature to obtain efficient desorption of CO_2_ in a short regeneration cycle (2 h). Figure 5a,b shows the capacity obtained for 20 wt % TEPA and PEI sorbents after 10 cycles of adsorption at −20 °C for 2 h and desorption at 60 and 50 °C, respectively, for 2 h as well. The average working capacity of the 20 wt % TEPA sample across the 10 cycles was 1.1 mmol g^–1^, whereas the average working capacity for the PEI sample was 0.71 mmol g^–1^. Both samples, especially the 20 wt % TEPA material, showed promising behavior over 10 cycles with capacities remaining stable across the 10 cycles. The average working capacities obtained were similar to the pseudo-equilibrium capacities obtained from Figures S3a and S4a. The stability and high working capacities obtained over multiple cycles show that the desorption temperatures chosen for the study were effective.

Similar measurements were performed for 40 wt % TEPA and PEI samples, where CO_2_ was absorbed at −20 °C and desorbed at 70 °C for TEPA and 60 °C for PEI, with these temperatures chosen based on the TPD results shown in Figures 2a and 4a. Both the samples were stable across the 10 cycles with very little reduction in capacities as seen in Figures S5 and S6. The average working capacity for the 40 wt % TEPA was 0.85 mmol g^–1^ which is surprisingly smaller than the working capacity for the 20 wt % TEPA sample even though the pseudo-equilibrium capacities show the opposite trend. The reason for the reversal in trend could be the longer timescales required for the 40 wt % TEPA sample to reach a pseudo-equilibrium, as seen in Figure 2a compared to the much quicker approach to equilibrium for the 20 wt % TEPA, which had less mass transfer limitations to CO_2_ diffusion. Hence, while 2 h of adsorption at −20 °C was enough for the 20 wt % sample to reach its pseudo-equilibrium capacity, the same is not true for the TEPA sample with a higher amine content.

Figures S7–S10 show the adsorption–desorption cycles for the 20 and 40 wt % TEPA and PEI sorbents with adsorption at 25 °C. All of the samples were stable across the 5 cycles at 25 °C (detailed discussion in Section S5). Overall, all of the sorbents with different amine content for both PEI and TEPA maintained their CO_2_ uptakes across the cycles tested at the different adsorption temperatures (25 and −20 °C). For the case of adsorption at 25 °C, the overall total temperature swing was 35–45 °C (desorption at 60 or 70 °C) while the swing was expectedly greater for the adsorption at −20 °C at 70–80 °C.

### 3.4 CO_2_ Adsorption under Humid Conditions in a Fixed Bed System

Ambient air always carries some moisture, the content of water vapor depending on the temperature of the air and location. The simultaneous adsorption of CO_2_ and H_2_O from the air onto the amine-based sorbents plays a critical role in determining the overall uptake of CO_2_.^16,69−71^ Reports have shown that the co-adsorption of water vapor onto the amine sites can enhance the CO_2_ adsorption due to the formation of bicarbonate or carbamate ion pairs, which enhances the amine efficiency of the sorbent.^16,69,70,72−74^ While cold air carries much less water vapor than hot air, the presence of even small amounts of moisture can affect the performance of the amine-based sorbents. Hence, it is imperative to investigate the effect of water vapor on the CO_2_ uptake performances of the amine-incorporated γ-Al_2_O_3_ samples.

**Figure 6:**
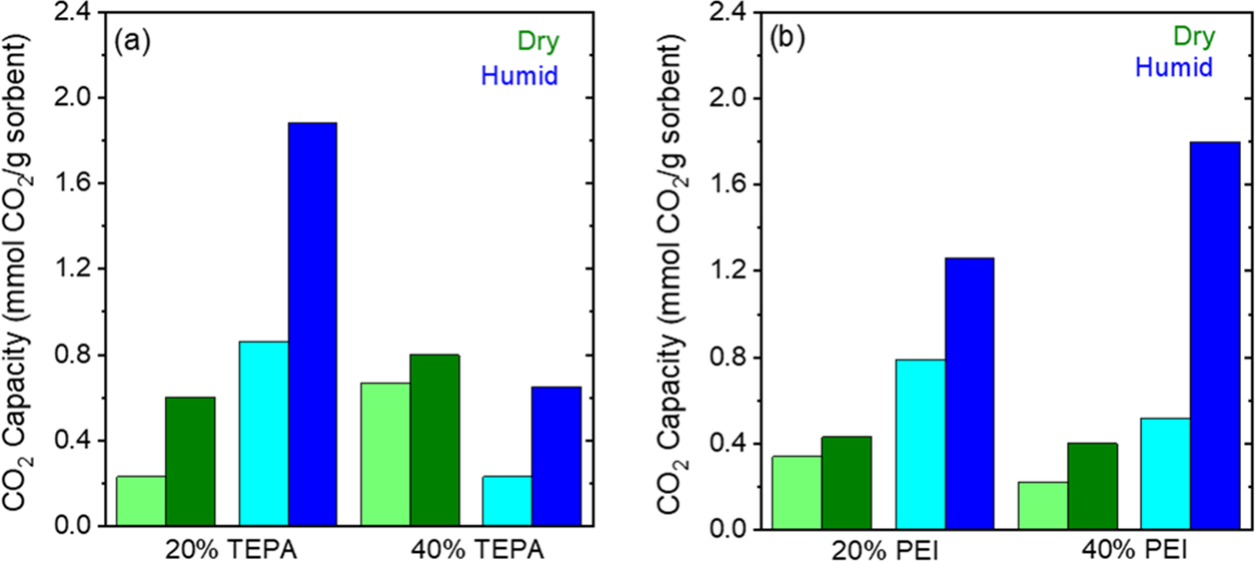
Breakthrough (*C*/*C*_0_ = 0.05, represented by lighter colors) and pseudo-equilibrium (*C*/*C*_0_ = 0.95, represented by darker colors) CO_2_ capacities under humid conditions (70% RH) at −20 °C and 400 ppm CO_2_ for (a) TEPA- and (b) PEI-impregnated γ-Al_2_O_3_ materials (measured in a fixed bed setup).

**Figure 7:**
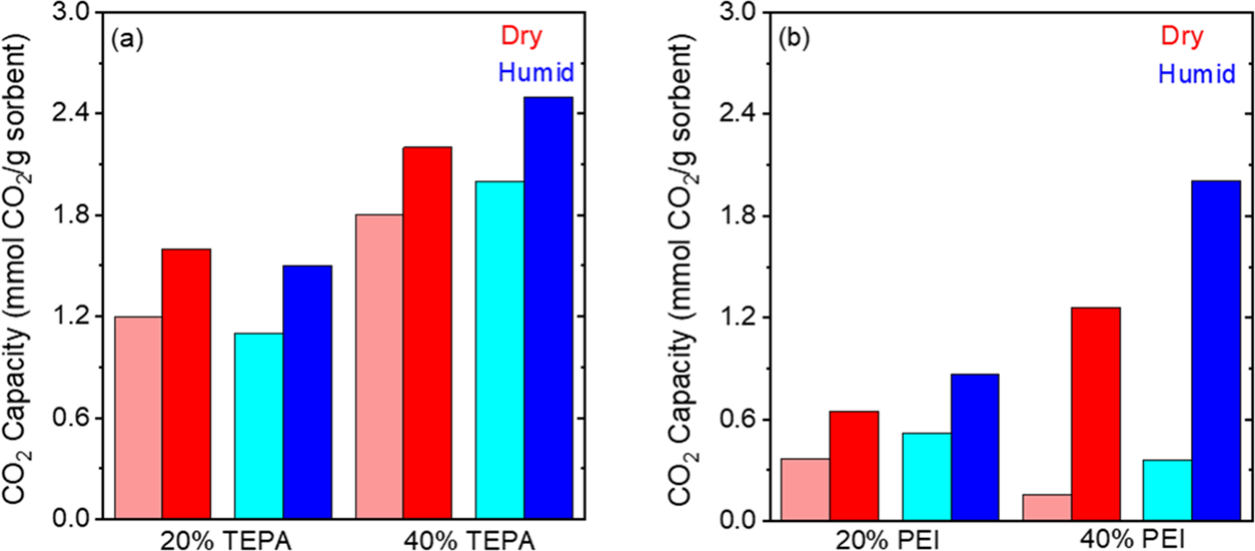
Breakthrough (*C*/*C*_0_ = 0.05, represented by lighter colors) and pseudo-equilibrium (*C*/*C*_0_ = 0.95, represented by darker colors) CO_2_ capacities under humid conditions (70% RH) at 25 °C and 400 ppm CO_2_ for (a) TEPA- and (b) PEI-impregnated γ-Al_2_O_3_ materials (measured in a fixed bed setup).

Figures 6 and 7 show that the presence of water vapor (RH 70%, 840 ppm at −20 °C, 13 970 ppm at 25 °C) improves the breakthrough (calculated at 5% *C*_0_) and pseudo-equilibrium CO_2_ capacities (calculated at 95% *C*_0_) for most of the samples (20 and 40 wt % TEPA and PEI sorbents) at the two temperature conditions. Figure S11a shows that the CO_2_ breakthrough time was longer for the humid conditions compared to the dry conditions for the 20 wt % TEPA sample at −20 °C. The longer breakthrough time leads to significantly higher breakthrough capacities as seen from Figure 6a. The breakthrough capacity for the 20 wt % TEPA sample increases from 0.23 to 0.86 mmol g^–1^, while the pseudo-equilibrium capacity increases from 0.6 to 1.8 mmol g^–1^. Even a small quantity of water at −20 °C doubles the CO_2_ uptake of 20 wt % TEPA. The amine efficiency, shown in Table 2, increases from 0.09 to 0.31. Previous works have attributed the enhancement in the CO_2_ capture performance in the presence of humidity to the greater formation of carbamate/carbamic acid species arising due to freeing of amines from the pore walls of the support^70,75,76^ or due to the formation of bicarbonate species.^77−79^ For the 40 wt % TEPA at −20 °C, the breakthrough capacity decreases from 0.65 to 0.23 mmol g^–1^; however, the pseudo-equilibrium capacity decreases only by ∼20%. Hence, CO_2_ capacity trends reverse under humid conditions for the 20 and 40 wt % TEPA samples. The initial rapid increase in the breakthrough curve followed by a slow approach to pseudo-equilibrium (Figure S12a) suggests that the higher amine loading restricts the effective diffusion of CO_2_ through the film of adsorbed water (since samples were prehumidified before CO_2_ exposure).

**Table 2:** Summary of Pseudo-Equilibrium CO_2_ Uptakes and Amine Efficiencies at −20 °C Obtained from Breakthrough Curves under Dry and Humid Conditions

	pseudo-equilibrium CO_2_ capacity (mmol CO_2_) (g sorbent)^−1^	amine efficiency (mol CO_2_) (mol N)^−1^	pseudo-equilibrium CO_2_ capacity (mmol CO_2_) (g sorbent)^−1^	amine efficiency (mol CO_2_) (mol N)^−1^
sample	dry		humid	
40 wt % PEI-Al_2_O_3_	0.4	0.03	1.8	0.12
20 wt % PEI-Al_2_O_3_	0.43	0.06	1.26	0.17
40 wt % TEPA-Al_2_O_3_	0.8	0.08	0.65	0.065
20 wt % TEPA-Al_2_O_3_	0.6	0.09	1.88	0.31

The presence of water vapor also has a positive effect on the CO_2_ capacities of PEI-impregnated γ-Al_2_O_3_ samples at −20 °C, as shown in Figure 6b. The breakthrough capacities for the 20 wt % PEI increase by ∼50%, while the pseudo-equilibrium capacity triples from 0.43 to 1.26 mmol g^–1^ when exposed to 70% RH at −20 °C. The 40 wt % PEI sample also sees an enhancement in the breakthrough capacity (from 0.22 to 0.52 mmol g^–1^). The increase in the pseudo-equilibrium capacity for the 40 wt % material is especially significant, with the capacity increasing from 0.4 to 2 mmol g^–1^, which is a 5-fold increase. The amine efficiency also increases from 0.06 to 0.17, as seen in Table 2. These results indicate that both 20 wt % TEPA with a capacity of 1.8 mmol g^–1^ and the 40 wt % PEI with a capacity of 2 mmol g^–1^ at 70% relative humidity at −20 °C are both promising materials for DAC operation under cold conditions.

The effects of humidity (70% RH) at 25 °C are less pronounced on the TEPA-impregnated sorbents compared to −20 °C. The breakthrough and pseudo-equilibrium capacities increase from 1.8 and 2.2 mmol g^–1^ to 2 and 2.5 mmol g^–1^, respectively (Figure 7a and Table 2), for the 40 wt % sorbent. While there is a 10–15% increase in the capacities, the enhancements in capacities due to humidity effects are much smaller at 25 °C compared to those observed at −20 °C for the TEPA samples. On the other hand, the effect of humidity on PEI samples at 25 °C is more pronounced than the TEPA samples. The breakthrough curves (Figures S13b and S14b) show that the presence of water vapor slows down the breakthrough time for both the 20 and 40 wt % PEI samples. The breakthrough capacity for the 20 wt % PEI sample increased slightly from 0.37 to 0.53 mmol g^–1^ while the pseudo-equilibrium capacities increased from 0.65 to 0.87 mmol g^–1^. The changes are much larger for the 40 wt % PEI samples, with the breakthrough capacity doubling from 0.16 to 0.36 mmol g^–1^ while the pseudo-equilibrium capacities increased from 1.26 to 2 mmol g^–1^. The amine efficiencies also increased from 0.08 to 0.14, as summarized in Table 3.

**Table 3:** Summary of Pseudo-Equilibrium CO_2_ Uptakes and Amine Efficiencies at 25 °C Obtained from Breakthrough Curves under Dry and Humid Conditions

	pseudo-equilibrium CO_2_ capacity (mmol CO_2_) (g sorbent)^−1^	amine efficiency (mol CO_2_) (mol N)^−1^	pseudo-equilibrium CO_2_ capacity (mmol CO_2_) (g sorbent)^−1^	amine efficiency (mol CO_2_) (mol N)^−1^
sample	dry		humid	
40 wt % PEI-Al_2_O_3_	1.26	0.08	2	0.14
20 wt % PEI-Al_2_O_3_	0.6	0.08	0.87	0.12
40 wt % TEPA-Al_2_O_3_	2.2	0.22	2.5	0.25
20 wt % TEPA-Al_2_O_3_	1.6	0.27	1.5	0.25

The results describing the effects of humidity vary with the amine content, adsorption temperatures, and amine identity as well, which is a departure from the consistent trends observed for PEI and TEPA under dry conditions. Generally, an increase in CO_2_ uptake is observed for both 20 and 40 wt % PEI samples at both the adsorption temperatures in the presence of humidity. Branched PEI contains tertiary amines that are not active for CO_2_ adsorption under dry conditions. However, in the presence of H_2_O, tertiary amines can form bicarbonates, in principle, contributing to an increase in CO_2_ capture. This hypothesis is consistent with previous report where tertiary amines, grafted onto SBA-15, showed a pronounced improvement in CO_2_ capacities due to the formation of bicarbonates under humid conditions.^73^ Hence, with the exposure to H_2_O, the number of sites “active” for CO_2_ capture may increase more significantly for PEI than TEPA, which contains only primary and secondary amines. An alternate hypothesis is that the longer PEI chains are more sensitive to water disrupting amine–amine hydrogen-bonding networks, and water more effectively lubricates the chains of PEI than TEPA. For the 20 wt % TEPA sample, it is likely that the formation of carbamate ions is enhanced at −20 °C in the presence of moisture.^49^ At higher loadings, diffusion limitations due to high pore filling (∼70%) coupled with frozen/arrested chains may be responsible for the lowered CO_2_ capacities.

**Figure 8:**
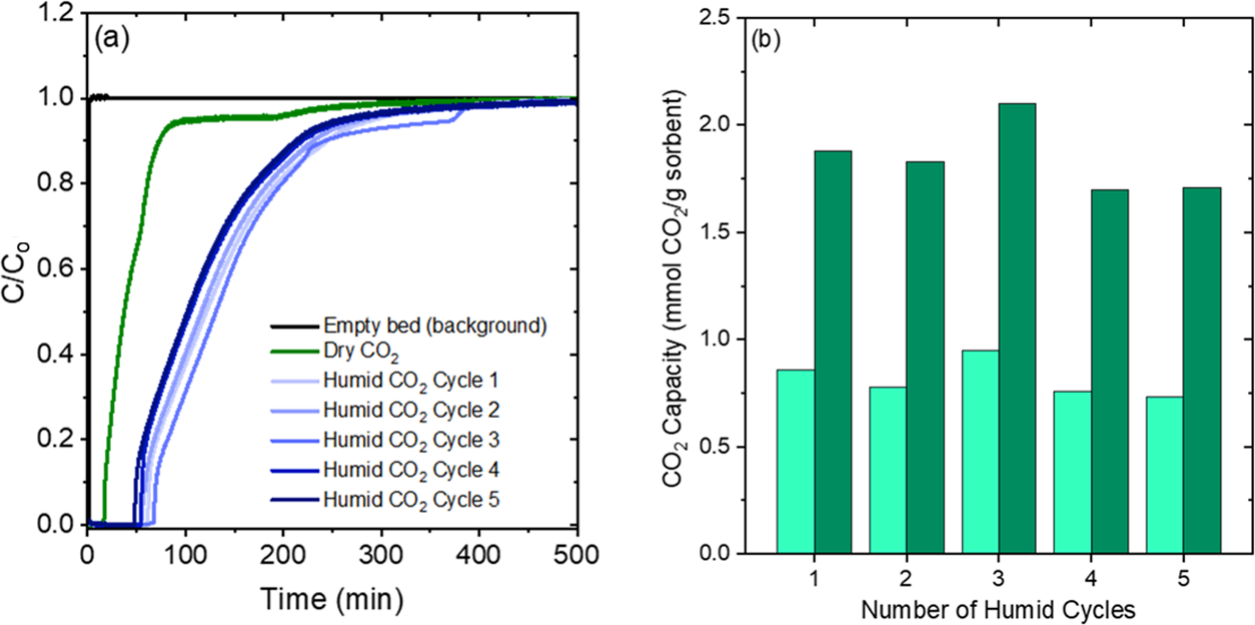
(a) Humid (70% RH) CO_2_ breakthrough curves at −20 °C for 20 wt % TEPA-impregnated γ-Al_2_O_3_ material. (b) Breakthrough (*C*/*C*_0_ = 0.05, light green) and pseudo-equilibrium (*C*/*C*_0_ = 0.95, dark green) CO_2_ capacities under humid conditions (70% RH) at −20 °C (measured in a fixed bed setup).

CO_2_ adsorption–desorption cyclic tests under humid conditions (70% RH at −20 °C) with a regeneration temperature of 60 °C (2 h) were conducted with the 20 wt % TEPA-impregnated γ-Al_2_O_3_ sample to evaluate the stability of the material under humid conditions. A prehumidifying step at the adsorption temperature was performed before each cycle, since the adsorbed water is also removed during the regeneration step, to ensure that the sorbent was fully saturated with water prior to CO_2_ adsorption over five consecutive cycles. This represents the wettest a sample might be at a given relative humidity in the absence of impacts from steam-stripping desorption, should that be the mode of desorption deployed at scale. Figure 8a shows that the CO_2_ breakthrough times for the five cycles were very similar, and both the breakthrough and pseudo-equilibrium capacities of each humid cycle were close in value across the 5 cycles as seen from Figure 8b, suggesting that the adsorbent was stable under the sub-ambient temperature, humid conditions. The overall breakthrough and pseudo-equilibrium capacities obtained were 0.72 and 1.6 mmol g^–1^. These results indicate that the 20 wt % TEPA-impregnated γ-Al_2_O_3_ powders can be effectively regenerated at 60 °C.

The results illustrated in this section show that humidity is a positive influence on the CO_2_ uptake for both the amine-based sorbents at low and moderate adsorption temperatures. The 20 wt % TEPA and 40 wt % PEI samples show high CO_2_ capacities at both −20 and 25 °C. The 20 wt % TEPA sample exhibits the highest amine efficiencies at all of the conditions across all samples and stability across multiple cycles, making it a worthy contender for DAC operation.

## 4 Future Directions and Study Limitations

The promising performance of PEI- and TEPA-impregnated powder sorbents (γ-Al_2_O_3_ in this study, and MIL-101(Cr) in previous studies^40,49^) at ambient and sub-ambient conditions, under both dry and humid conditions, provides preliminary data that can be used to further optimize these sorbents. Powder sorbents are ideal for lab-scale applications but not for industrial applications when pressure drop is a key performance metric, as in DAC. Contactors such as monoliths and fibers are more suitable for large-scale industrial applications. Alumina monoliths impregnated with PEI have shown promise in earlier works at 30 °C.^80^ Such materials need to be evaluated at a wider range of conditions (adsorption temperature, humidity) to optimize performance.

Another important aspect to be considered is the long-term stability of the amines. Both PEI and TEPA will degrade in the presence of O_2_.^81,82^ In this study, CO_2_ uptakes decreased by 15–20% for the PEI- and TEPA-impregnated samples after storage over a span of 6 months, with TEPA samples losing more activity than PEI. It is vital to understand the mechanisms of degradation of the amines over long timescales since it is important that the sorbents preserve their high adsorption performances over multiple months and also investigate alternative amines that are most resistant to aging effects such as poly(propyleneimine) (PPI).^83,84^ Significant efforts are being expended into understanding the degradation mechanisms for PEI-impregnated samples.^85−87^ Lesser attention has been directed toward TEPA samples, though the similarity of repeat units may make extrapolation from PEI possible.^88^ However, as seen from the study here, support identity, amine structure, and humidity all affect performance. Hence, it is likely that degradation mechanisms will also be sensitive to these factors (e.g., the presence of catalytic oxidation sites in some supports or amine samples),^89^ and it will be important to design carefully controlled studies to study degradation and devise strategies to combat this phenomenon and develop optimum materials for DAC applications.

Finally, it is expected that TEPA may not be a viable amine in DAC sorbents for moderate- to high-temperature operation. Its significant volatility (relative to PEI) makes it better suited for consideration in moderate or cold climates.

## 5 Conclusions

In this study, γ-Al_2_O_3_ was infused with branched PEI and TEPA at different loadings (10, 20, and 40 wt %) and the obtained sorbents were evaluated for CO_2_ capture at sub-ambient (−20 °C) and ambient (25 °C) dry and humid adsorption conditions. At 25 °C, CO_2_ uptake for both PEI and TEPA-impregnated samples increases with an increase in amine content. The 40 wt % TEPA material demonstrated the highest CO_2_ capacity at 1.8 mmol CO_2_/g sorbent while the 40 wt % PEI sample showed an uptake of 1 mmol g^–1^. When the adsorption temperature was lowered to −20 °C, CO_2_ capacities decreased for both 20 and 40 wt % PEI- and TEPA-impregnated γ-Al_2_O_3_ samples while they increased marginally for the 10 wt % loadings for both the amine sorbents. At the sub-zero temperature of adsorption, the high pore filling of the higher weight loading samples coupled with the arrested amine chains lead to pore-blocking that hindered the diffusion of CO_2_ through the pores, leading to reduced CO_2_ capacities of higher amine loading samples at −20 °C. Initial adsorption rates at −20 °C for both 40 and 20 wt % were comparable to those of 25 °C. Insights provided by the TPD data suggested that CO_2_ was strongly bound to the amines at the higher weight loadings for both PEI and TEPA, due to a greater concentration of free amines present at these higher loadings. When PEI and TEPA loadings were decreased to 10 wt %, the interaction of CO_2_ with the amine moieties became weaker because of the absence of significant amounts of free amines since a greater fraction of the amines interact strongly with the walls of the support compared to the higher loadings. Temperature swing adsorption–desorption cyclic tests on 20 and 40 wt % TEPA and PEI samples showed that all of the samples maintained their capacities across the cycles tested. These results showed that both the PEI- and TEPA-impregnated samples were stable at both −20 and 25 °C across multiple cycles under dry conditions. The effect of humidity was also evaluated on the sorbents since water vapor is ubiquitous at all temperatures and can greatly influence CO_2_ uptakes. In the presence of 70% RH at both −20 and 25 °C, CO_2_ capacities increased for both the 20 and 40 wt % PEI samples. In contrast, results for TEPA were mixed, with CO_2_ capacities increasing for 20 wt % TEPA at −20 °C and 40 wt % TEPA at 25 °C. The 20 wt % TEPA-impregnated γ-Al_2_O_3_ sample showed the best pseudo-equilibrium capacities (1.8 and 1.5 mmol g^–1^), with the highest amine efficiencies at both −20 and 25 °C, respectively, followed by 40 wt % PEI samples. These results show that TEPA- and PEI-infused γ-Al_2_O_3_ samples show promise as DAC sorbents across a wide range of temperature conditions. While crucial work still needs to be conducted to optimize these sorbents so that they may become industrially viable, these preliminary results help expand the scope of operating conditions for DAC, which may become an important negative emissions technology alongside.

## Supporting Information Available

Structures of amines used in the study; characterization of sorbents; effect of chain length on CO_2_ capacities; CO_2_ adsorption and desorption profiles; temperature swing adsorption–desorption cycles from TGA/DSC; and breakthrough curves from humid experiments in fixed bed (PDF)
