# Supplementary material for: Direct Air Capture of CO2 Using Amine/Alumina Sorbents at Cold Temperature
Source: ACS Environ Au. 2023 Jun 29;3(5):295–307. doi: 10.1021/acsenvironau.3c00010 (PMC10515709; doi:10.1021/acsenvironau.3c00010)
Supplement: Supplementary file 1 — vg3c00010_si_001.pdf [file vg3c00010_si_001.pdf]

Supporting Information

for

Direct Air Capture of CO_2 using Amine/Alumina Sorbents at Cold Temperature

Pranjali Priyadarshini, Guanhe Rim, Cornelia Rosu, MinGyu Song, Christopher W. Jones*

School of Chemical & Biomolecular Engineering, Georgia Institute of Technology, 311 Ferst Dr., Atlanta, Georgia 30332-0100, United States

Corresponding Author

* E-mail: cjones@chbe.gatech.edu

S1. Structures of amines used in the study

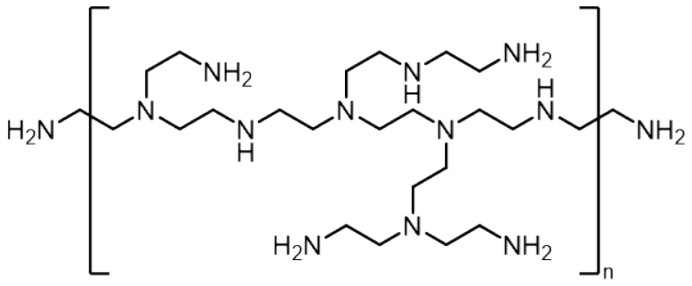

Branched poly(ethyleneimine)

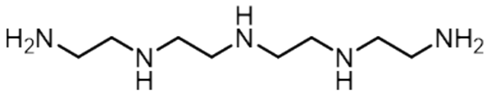

Tetraethylenepentamine (TEPA)[I]

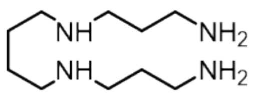

Spermine

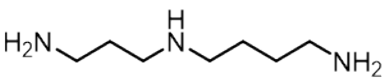

Spermidine

Scheme S1: Structure of the amines impregnated into the pores of γ-Al_2O_3. [I] Note, TEPA exists as a mixture of compounds

S2. Characterization of sorbents

Table S1: Physical characteristics of the sorbents

Sample	Theoretical Amine Content (%)	Actual Amine Content[a] (%)	BET Surface Area (m^2 g^{-1})	Pore Volume[b] (cm^3 g^{-1})	Pore filling (%)
Al_2O_3	-	-	124	1	-
PEI-Al_2O_3	40	35.7	24.5	0.28	72
PEI-Al_2O_3	20	18.3	68.3	0.58	42
PEI-Al_2O_3	10	9.2	106.2	0.7	30
TEPA-Al_2O_3	40	37.5	26.9	0.35	65
TEPA-Al_2O_3	20	23	53.9	0.66	34
TEPA-Al_2O_3	10	11.4	97.8	0.77	23

[a] determined by combustion TGA
[b] evaluated at P/P_o=0.99

Table S1 shows that the impregnation procedure of PEI and TEPA decreases the total pore volume and Brunauer-Emmett-Teller (BET) surface area of the γ-Al_2O_3 support. This indicates the successful incorporation of the amines into the pores of γ-Al_2O_3. However, some deposition of the amines on the outer surface of the support cannot be ruled out, especially at the higher amine loadings. As expected, the BET surface areas decrease with an increase in the amine content while the pore filling increases.

S3. Effect of chain length on CO_2 capacities

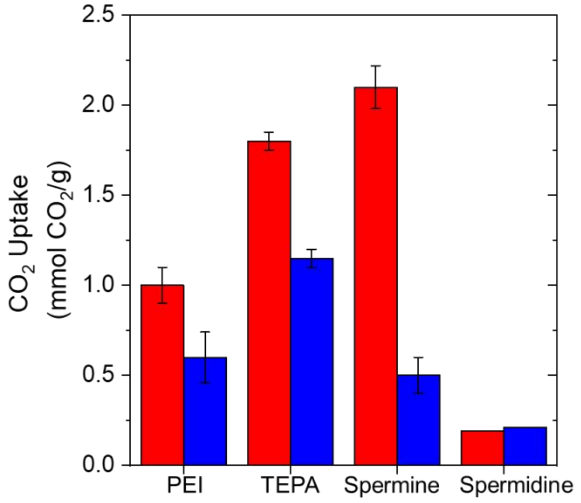

Figure S1: CO_2 uptakes for 40 wt.% amine (PEI, TEPA, Spermine and Spermidine)-impregnated γ-Al_2O_3 sorbents at 25 °C and -20 °C and 400 ppm CO_2.

Apart from PEI and TEPA, spermine and spermidine (**Scheme S1**) were also impregnated into the pores of alumina (40 wt.%) and their CO_2 capacities were measured at 25 °C and -20 °C to understand the effect of varying chain lengths on the CO_2 uptakes. At 25 °C, the spermine-impregnated γ-Al_2O_3 sample showed the highest CO_2 capacity among all the samples (~ 2 mmol/g), while spermidine showed the lowest capacity (~0.2 mmol/g) as seen in **Figure S1**. However, the CO_2 capacity for the spermine sample drastically reduced to 0.5 mmol/g while the capacity for the spermidine sample remained the same at -20 °C. Burnout experiments to determine the organic content in the γ-Al_2O_3 support showed that much less spermidine was present in the pores of alumina (< 10 wt.%). Both spermine and spermidine are more volatile than TEPA, while TEPA is more volatile than PEI.[1-2] Spermidine being highly volatile, escaped from the pores to a large extent during the drying of the sample in the rotary evaporator during synthesis. There was also significant loss of spermine during the activation of the sample before measurement, which may be the reason for the drastic decrease in the CO_2 uptakes at -20 °C. Since both the spermine- and spermidine-incorporated γ-Al_2O_3 sorbents displayed poor CO_2 capacities at -20 °C and are more volatile than TEPA, they were not considered as promising candidates for DAC applications at ambient or sub-ambient conditions and further exploration of their behavior is not reported.

S4. CO_2 adsorption and desorption profiles

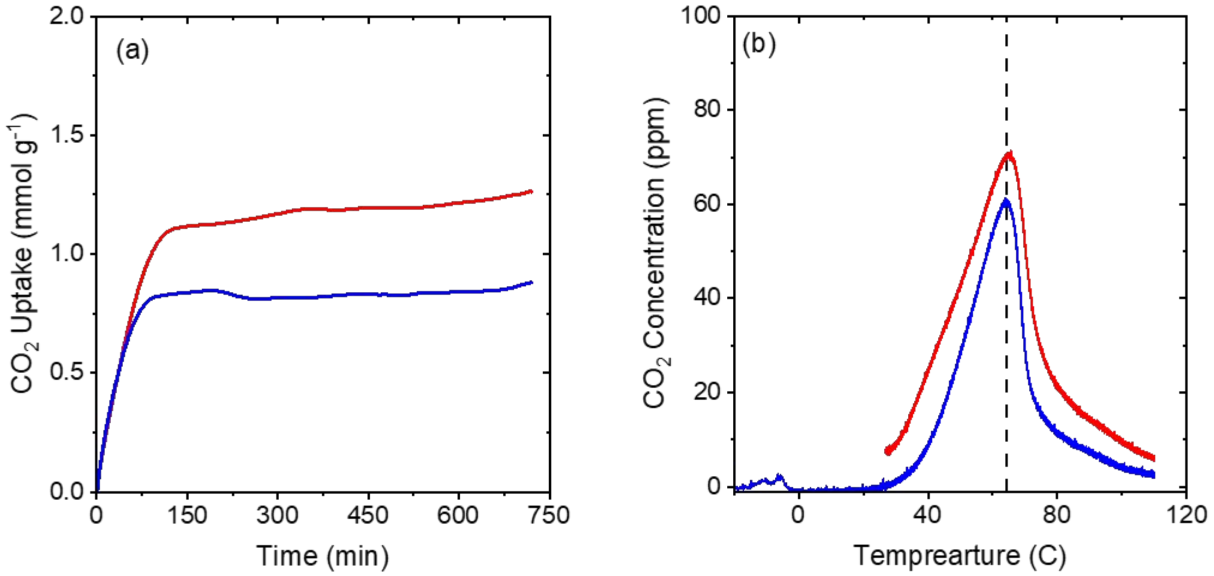

Figure S2. (a) CO_2 adsorption profile of 20 wt.% TEPA-impregnated γ-Al_2O_3 at 25 °C and -20 °C at 400 ppm CO_2 (balance He). (b) CO_2-TPD profiles after adsorption at 25 °C and -20 °C.

Figure S2a shows that trends for 20 wt.% TEPA samples are similar to the trends observed in **Figure 1** where the CO_2 capacities decrease with temperature, however to a lesser extent as compared to the 40 wt.% sample. The 20 wt.% sample reaches a pseudoequilibrium capacity unlike the 40 wt.% sample (Figure 2a) due to lower inhibition to CO_2 diffusion since the pore volume of the 20 wt.% sample is greater than the 40 wt.% sorbent. **Figure S2b** shows the desorption profile for CO_2 adsorption at 25 °C and -20 °C for the 20 wt.% TEPA sample. The trends are very similar to that of the 40 wt.% TEPA sample where the CO_2 desorption profile shows a small physisorbed CO_2 peak for the adsorption at -20 C below 0 °C and a peak at ~60 °C for both adsorption temperatures. Apart from this difference, the desorption peaks for the 20 wt.% sample look similar irrespective of the CO_2 adsorption temperature.

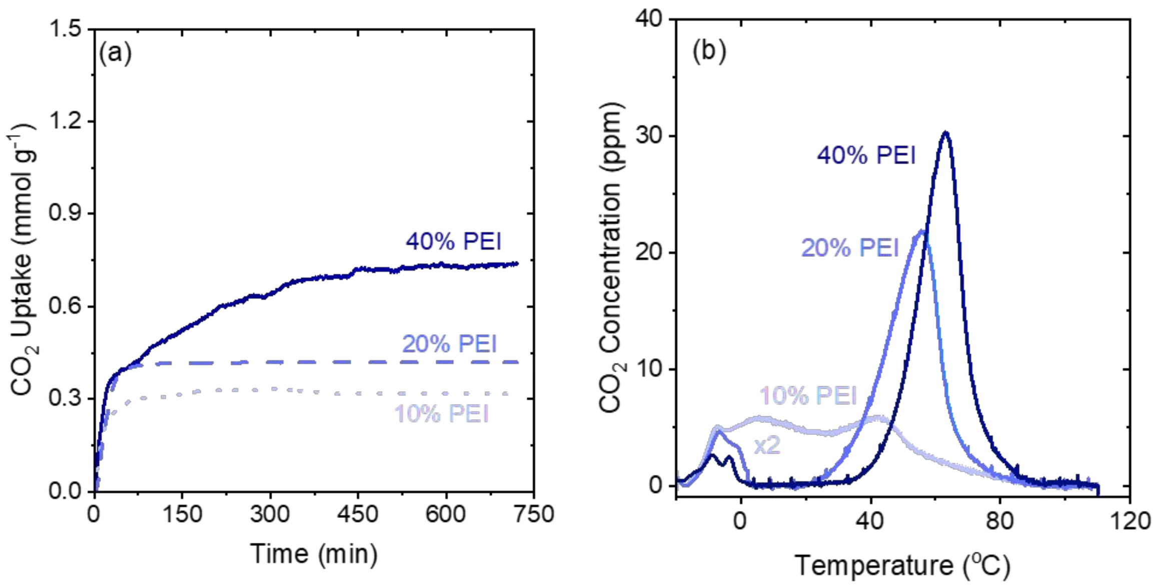

Figure S3. (a) CO_2 uptake profile of 40 wt.%, 20 wt.% and 10 wt.% PEI-impregnated γ-Al_2O_3 at -20 °C and 400 ppm CO_2 (balance He). (b) CO_2-TPD profiles after CO_2 adsorption at -20 °C. The CO_2 adsorption profiles at -20 °C are often wavy due to the injection of pulses of liquid nitrogen intermittently to maintain the temperature in the TGA/DSC.

Figure S3a shows that as the amine content in the sample decreases from 40 wt.% to 10 wt.%, the time taken to reach a pseudoequilibrium decreases. The quicker approach to pseudoequilibrium with lower amine loading happens for similar reasons as described for the TEPA samples. The pore volume occupied by the PEI decreases as the amine loading decreases, as seen from **Table S1**, which decreases the barriers to diffusion of CO_2, hence allowing a quicker approach to pseudoequilibrium. The trend for the desorption profiles mimics the trends for the TEPA samples. The desorption peak moves to a lower temperature as the amine content decreases, indicating that interaction of CO_2 with PEI weakens, which in turn implies that the interaction of the PEI chains with the walls has increased, on average. The differences in desorption profiles are especially obvious for the 10 wt.% PEI sample, which shows very broad peaks of CO_2 desorption. The peaks occurring at lower desorption temperatures indicate that the CO_2 interacts weakly with the amines. Reminiscent of TEPA samples, PEI would also mostly coat the walls of γ-Al_2O_3 at lower amine loadings, thus interacting more with the walls and less with CO_2, which manifests as lower desorption peak temperatures. On the other hand, after initially coating the walls of the support, PEI likely forms multilayers of aggregates at higher amine loadings,[3] which contain a greater fraction of free amines, thereby reducing the average degree of interaction with the walls of the support.

Table S2: Summary of adsorption rates for different sorbents at 25 and -20 °C

Sample	Initial Adsorption Rate 25 °C (mmol CO_2) (g sorbent)$^{-1}$min^{-1}	Initial Adsorption Rate -20 °C (mmol CO_2) (g sorbent)$^{-1}$min^{-1}
40 wt.% PEI-Al_2O_3	0.022	0.026
20 wt.% PEI-Al_2O_3	0.020	0.017
10 wt.% PEI-Al_2O_3		0.019
40 wt.% TEPA-Al_2O_3	0.012	0.011
20 wt.% TEPA-Al_2O_3	0.023	0.021
10 wt.% TEPA-Al_2O_3		0.025

S5. Temperature swing adsorption-desorption cycles from TGA/DSC

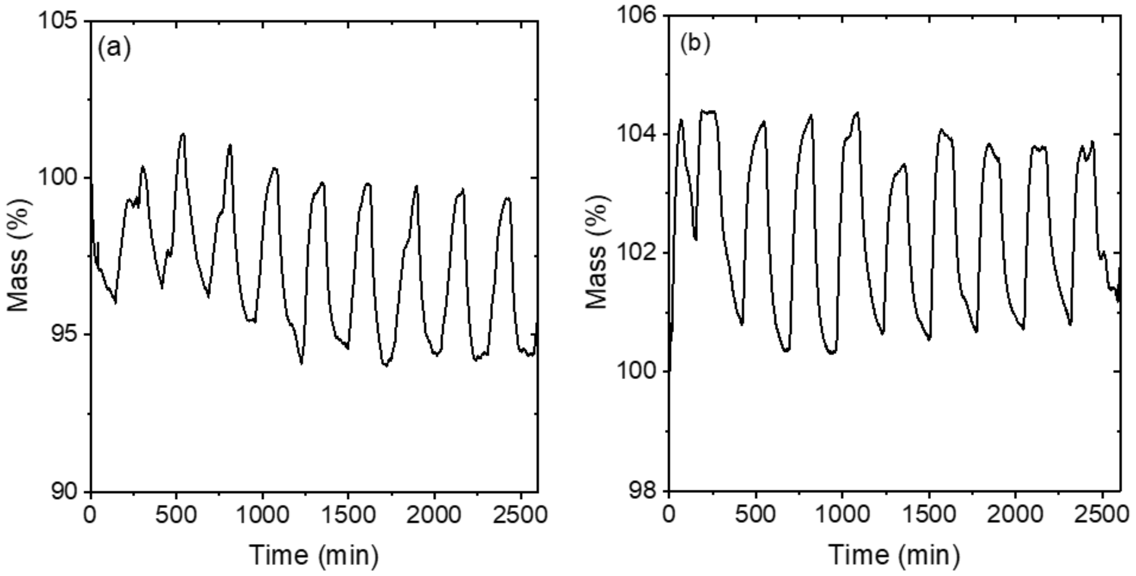

Figure S4. Sample mass change of (a) 20 wt.% TEPA and (b) 20 wt.% PEI impregnated γ-Al_2O_3 over 10 CO_2 adsorption and desorption cycles. CO_2 adsorption measurement was performed at -20 °C, 400 ppm CO_2 (balance He) for 2 h, and desorption at 60 °C for TEPA and 50 °C for PEI for 2 h. The CO_2 adsorption profiles at -20 °C are often wavy due to the injection of pulses of liquid nitrogen intermittently to maintain the temperature in the TGA/DSC

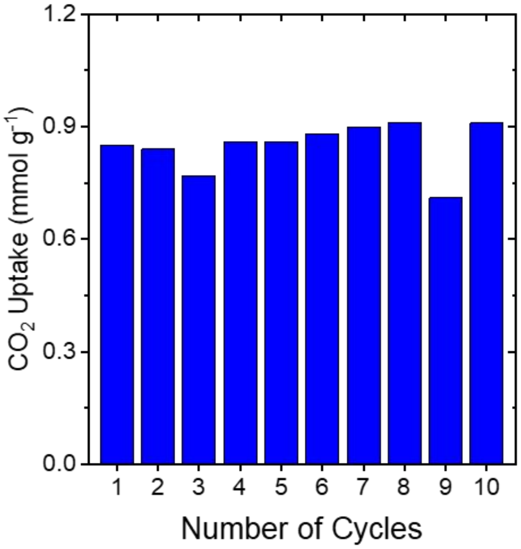

Figure S5: CO_2 adsorption working capacity across 10 adsorption-desorption cycles on 40 wt.% TEPA-impregnated γ-Al_2O_3 sample. CO_2 adsorption measurement was performed at -20 °C, 400 ppm CO_2 (balance He) for 2 h, and desorption at 70 °C for 2 h.

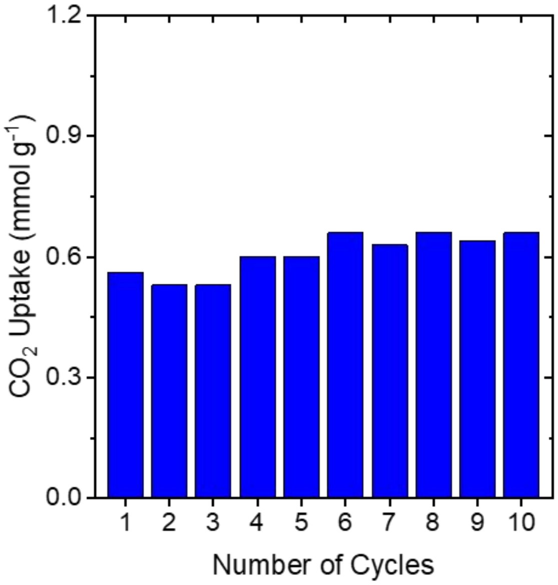

Figure S6: CO_2 adsorption working capacity across 10 adsorption-desorption cycles on 40 wt.% PEI-impregnated γ-Al_2O_3 sample. CO_2 adsorption measurement was performed at -20 °C, 400 ppm CO_2 (balance He) for 2 h, and desorption at 60 °C for 2 h.

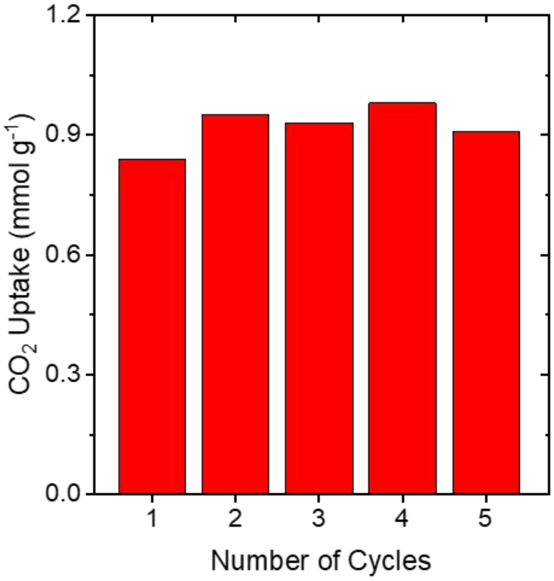

Figure S7: CO_2 adsorption working capacity across 5 adsorption-desorption cycles on 20 wt.% TEPA-impregnated γ-Al_2O_3 sample. CO_2 adsorption measurement was performed at 25 °C, 400 ppm CO_2 (balance He) for 2 h, and desorption at 60 °C for 2 h.

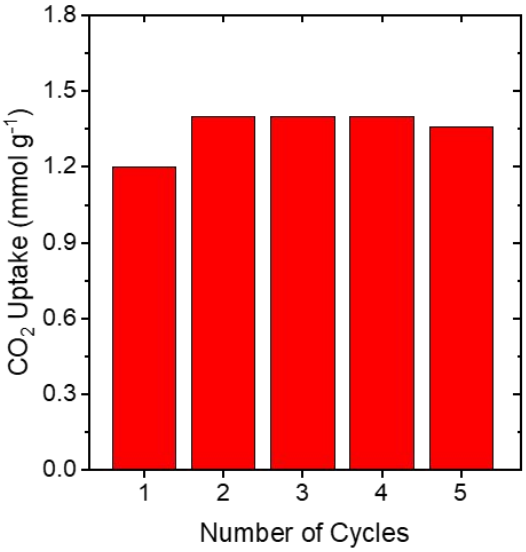

Figure S8: CO_2 adsorption working capacity across 5 adsorption-desorption cycles on 40 wt.% TEPA-impregnated γ-Al_2O_3 sample. CO_2 adsorption measurement was performed at 25 °C, 400 ppm CO_2 (balance He) for 2 h, and desorption at 70 °C for 2 h.

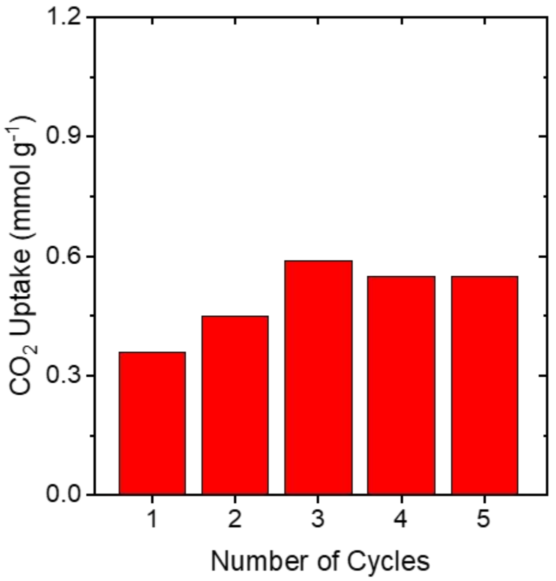

Figure S9: CO_2 adsorption working capacity across 5 adsorption-desorption cycles on 20 wt.% PEI-impregnated γ-Al_2O_3 sample. CO_2 adsorption measurement was performed at 25 °C, 400 ppm CO_2 (balance He) for 2 h, and desorption at 50 °C for 2 h.

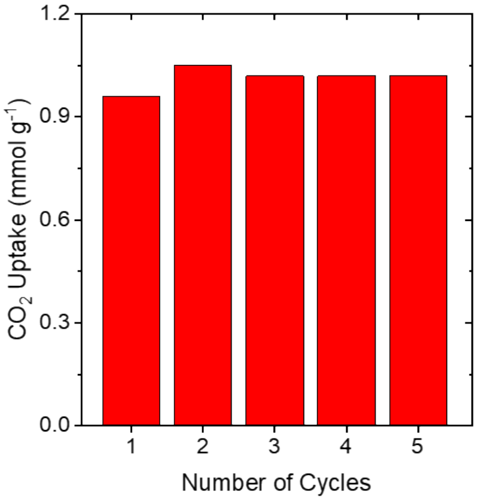

Figure S10: CO_2 adsorption working capacity across 5 adsorption-desorption cycles on 40 wt.% PEI-impregnated γ-Al_2O_3 sample. CO_2 adsorption measurement was performed at 25 °C, 400 ppm CO_2 (balance He) for 2 h, and desorption at 60 °C for 2 h.

The TPD results (**Section 3.1** and **S3**) show that, unlike MIL-101(Cr) materials incorporated with PEI and TEPA,[4] the desorption profiles for amine-based γ-Al_2O_3 sorbents are not significantly affected by the adsorption temperatures for the higher amine loading samples. Hence, for CO_2 adsorption at 25 °C, the same desorption conditions as those for adsorption at -20 °C were used for both PEI and TEPA. **Figures S7-S10** show the adsorption-desorption cycles for the 20 wt.% and 40 wt.% TEPA and PEI sorbents. All the samples were stable across the 5 cycles at 25 °C. The average working capacity of the 40 wt. % TEPA and 20 wt.% TEPA samples were 1.3 and 0.9 mmol/g, respectively. The 40 wt.% TEPA sorbent showed lower working capacity than the pseudoequilibrium capacity obtained from **Figure 2**, while the 20 wt.% TEPA showed a very similar working capacity to the pseudoequilibrium capacity. This is also due to the 40 wt.% TEPA sample suffering from slower adsorption rates after the initial rapid uptake (**Figure 2**) as compared to the faster adsorption rates for the 20 wt.% TEPA. The 40 wt.% PEI sample showed a working capacity of 1 mmol/g sorbent while the 20 wt.% PEI sorbent showed a working capacity of 0.5 mmol/g. Both values are close to the pseudoequilibrium capacities obtained from long timescale adsorption measurements.

S6. Breakthrough curves from humid experiments in fixed bed

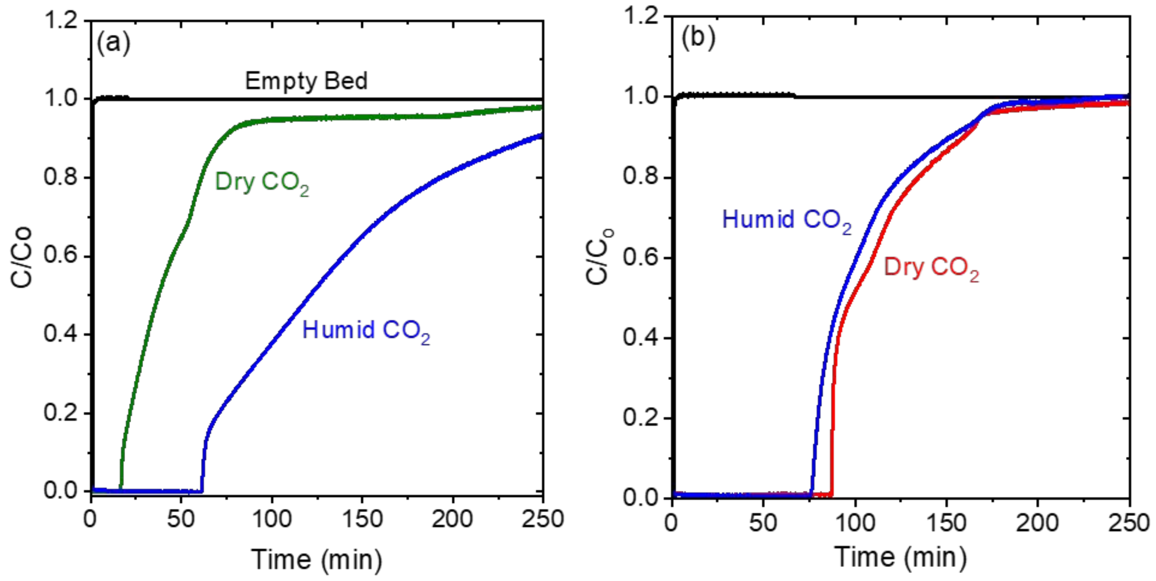

Figure S11. CO_2 breakthrough curves for dry and humid (70% RH) 400 ppm CO_2 on 20 wt.% TEPA-impregnated γ-Al_2O_3 powders in a fixed bed system at (a) −20 °C and (b) 25 °C.

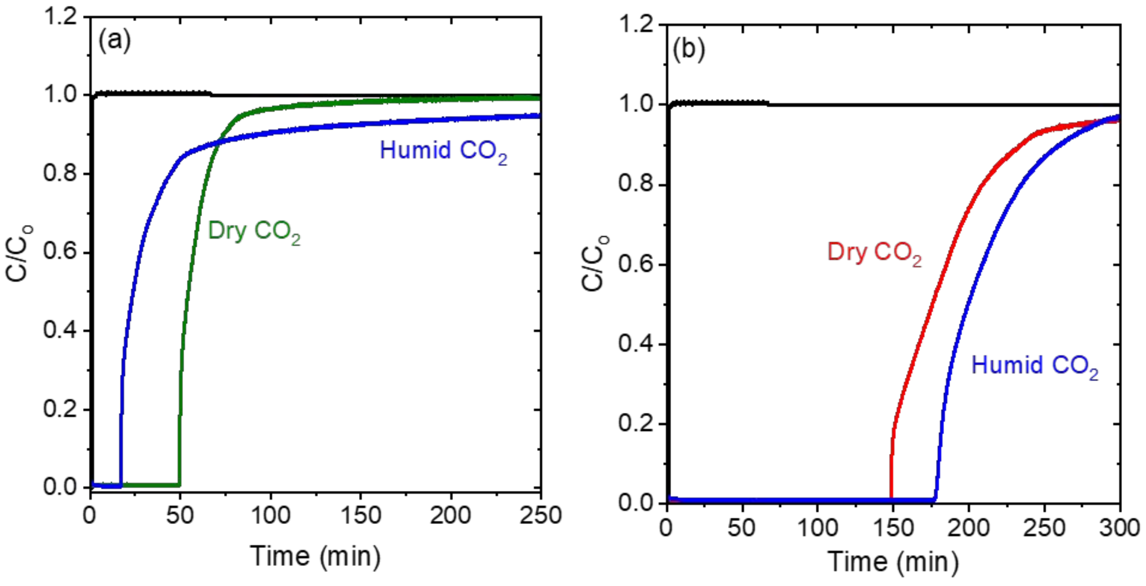

Figure S12. CO_2 breakthrough curves for dry and humid (70% RH) 400 ppm CO_2 on 40 wt.% TEPA-impregnated γ-Al_2O_3 powders in a fixed bed system at (a) −20 °C and (b) 25 °C.

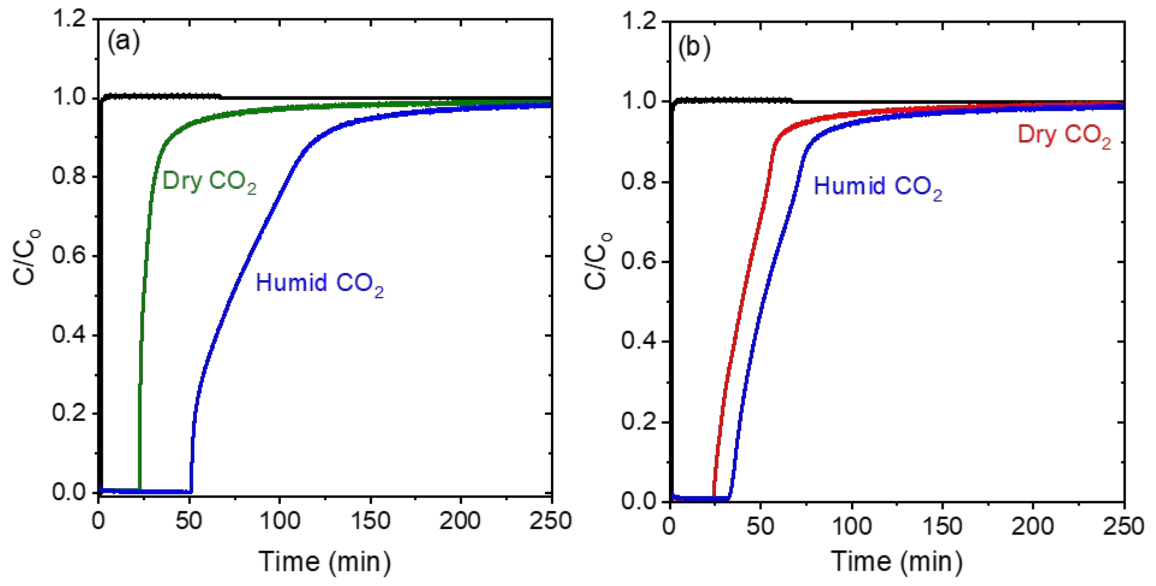

Figure S13. CO_2 breakthrough curves for dry and humid (70% RH) 400 ppm CO_2 on 20 wt.% PEI-impregnated γ-Al_2O_3 powders in a fixed bed system at (a) −20 °C and (b) 25 °C.

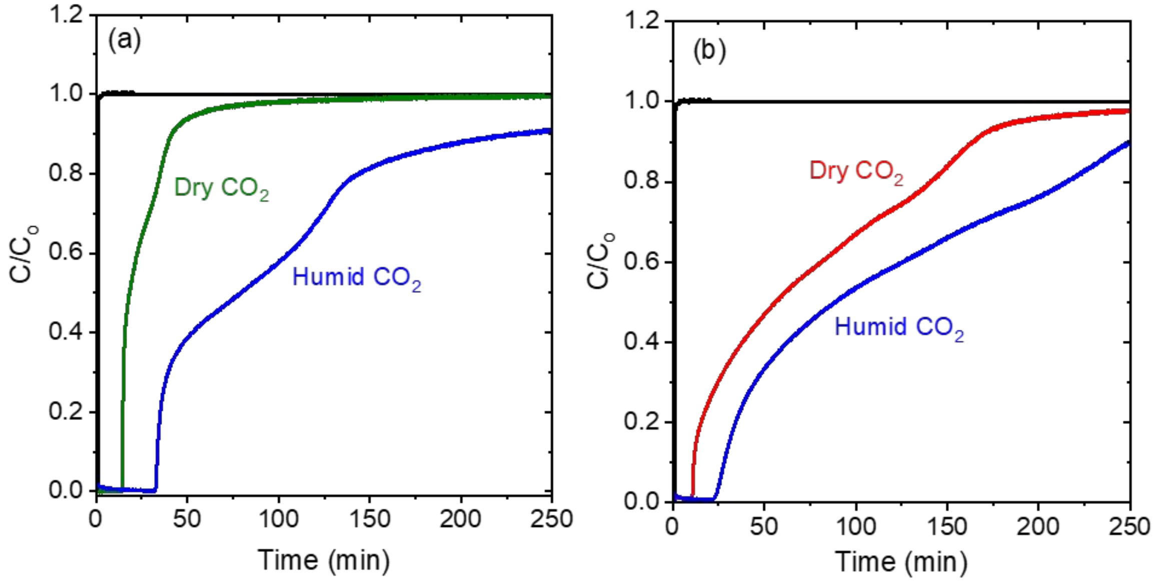

Figure S14. CO_2 breakthrough curves for dry and humid (70% RH) 400 ppm CO_2 on 40 wt.% PEI-impregnated γ-Al_2O_3 powders in a fixed bed system at (a) −20 °C and (b) 25 °C.

References

1. Ross, B. M.; Babay, S.; Ladouceur, C., The use of selected ion flow tube mass spectrometry to detect and quantify polyamines in headspace gas and oral air. *Rapid Commun. Mass Spectrom.* **2009,** *23* (24), 3973.
2. Zhang, X.; Zhang, S.; Qin, H.; We, W., Development of TRPN dendrimer-modified disordered mesoporous silica for CO_2 capture. *Mater. Res. Bull.* **2014,** *56*, 12.
3. Holewinski, A.; Sakwa-Novak, M. A.; Jones, C. W., Linking CO_2 sorption performance to polymer morphology in aminopolymer/silica composites through neutron scattering. *J. Am. Chem. Soc.* **2015,** *137* (36), 11749.
4. Rim, G.; Kong, F.; Song, M.; Rosu, C.; Priyadarshini, P.; Lively, R. P.; Jones, C. W., Sub-ambient temperature direct air capture of CO_2 using amine-impregnated MIL-101(Cr) enables ambient temperature CO_2 recovery. *JACS Au* **2022,** *2* (2), 380.
